# Effect of self-managed lifestyle treatment on glycemic control in patients with type 2 diabetes

**DOI:** 10.1038/s41746-022-00606-9

**Published:** 2022-05-11

**Authors:** Chinmay Dwibedi, Emelia Mellergård, Amaru Cuba Gyllensten, Kristoffer Nilsson, Annika S. Axelsson, Malin Bäckman, Magnus Sahlgren, Stephen H. Friend, Sofie Persson, Stefan Franzén, Birgitta Abrahamsson, Katarina Steen Carlsson, Anders H. Rosengren

**Affiliations:** 1 Department of Neuroscience and Physiology, Sahlgrenska Academy at the University of Gothenburg, Gothenburg, Sweden; 2 Faculty of Medicine, Lund University, Lund, Sweden; 3 Research Institutes of Sweden, Göteborg, Sweden; 4 Swedish Institute for Health Economics, Lund, Sweden; 5 Department of Psychiatry, University of Oxford, Oxford, United Kingdom; 6 RegisterCentrum Västra Götaland, Göteborg, Sweden; 7 Health Metrics, Department of Public Health and Community Medicine, University of Gothenburg, Gothenburg, Sweden

**Keywords:** Type 2 diabetes, Lifestyle modification

## Abstract

The lack of effective, scalable solutions for lifestyle treatment is a global clinical problem, causing severe morbidity and mortality. We developed a method for lifestyle treatment that promotes self-reflection and iterative behavioral change, provided as a digital tool, and evaluated its effect in 370 patients with type 2 diabetes (ClinicalTrials.gov identifier: NCT04691973). Users of the tool had reduced blood glucose, both compared with randomized and matched controls (involving 158 and 204 users, respectively), as well as improved systolic blood pressure, body weight and insulin resistance. The improvement was sustained during the entire follow-up (average 730 days). A pathophysiological subgroup of obese insulin-resistant individuals had a pronounced glycemic response, enabling identification of those who would benefit in particular from lifestyle treatment. Natural language processing showed that the metabolic improvement was coupled with the self-reflective element of the tool. The treatment is cost-saving because of improved risk factor control for cardiovascular complications. The findings open an avenue for self-managed lifestyle treatment with long-term metabolic efficacy that is cost-saving and can reach large numbers of people.

## Introduction

Lifestyle-related diseases such as type 2 diabetes are major causes of death and disability and put strains on health expenditures world-wide^1^. Structured lifestyle treatment is currently limited by the associated need for large healthcare resources. Digital tools could enable clinical utility but most solutions require coaching, intensified healthcare activities or user fees, which hinder broad application. In view of the escalating numbers of afflicted people, also in low- and middle-income countries, it is necessary to find new ways to support lifestyle self-management and complement drug treatment and resource-intense lifestyle programs^2–4^. This is, however, currently hampered by several important knowledge gaps^5^.

First, data on long-term effects (>6 months) are scarce^5–8^. Recent meta-analyses have shown declining metabolic response to digital lifestyle interventions after six months and most studies to date lack investigation of the effective behavior change techniques and have applied only limited measures to adjust for potential confounders^5–11^. Second, digital tools are often combined with coaching or intensified healthcare contacts, making it difficult to specifically assess the effect of the digital component and apply the results to a broad range of settings^5,8–11^. In fact, one of the major limitations to structured lifestyle support, whether traditional or digital, is that it currently requires increased efforts by healthcare professionals or costs for the individual patient, and less than one out of ten individuals with diabetes take part in such programs^3^. Third, studies from individuals at risk for diabetes have indicated an impact of BMI and insulin resistance on the effect of exercise, but how the individual pathophysiology influences the response to lifestyle treatment in patients with manifest type 2 diabetes remains unknown^12,13^. This is pertinent to address, especially in light of recent data, emphasizing the clinical variability of type 2 diabetes and highlighting four clusters of patients with different disease characteristics^14^. Such information could potentially help tailor lifestyle programs to those who are most likely to benefit^8^.

Here we developed and evaluated a self-managed lifestyle tool that is scalable and does not require additional healthcare resources. We hypothesized that HbA1c, reflecting long-term blood glucose, would decrease in patients with type 2 diabetes exposed to the tool, both compared to randomized wait list controls and matched controls. We also hypothesized that the tool would be particularly effective in one of the recently identified clusters, termed Mild Obesity-related Diabetes (MOD), which represents obese insulin-resistant patients and encompasses 22–30% of individuals with type 2 diabetes^14^. Finally, we aimed to assess the effective elements of the treatment by natural language processing using machine-learning.

## Results

### Development of the tool

**Fig. 1:**
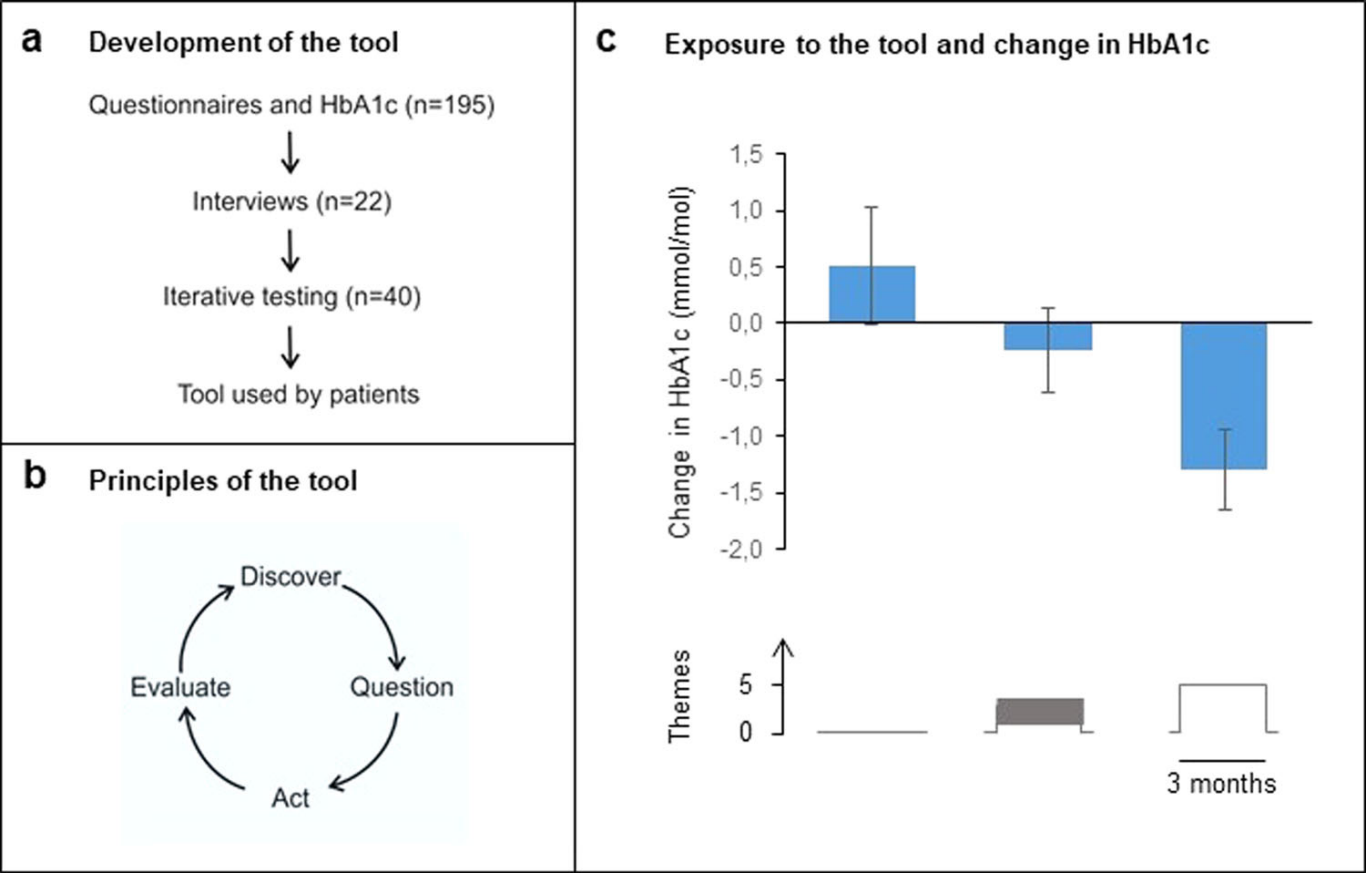
Outline of the intervention tool. Development (**a**), principles (**b**) and effect of exposure to the tool (**c**). **a** shows the step-wise development of the tool, commencing with longitudinal analyses of psychological factors associated with HbA1c progression in 195 patients with T2D. This was followed by semi-structured interviews on unmet needs in lifestyle management and expectations of a digital lifestyle tool. The tool underwent repeated testing and modification based on feedback from patients and healthcare professionals on content, language, usability, and relevance. **b** depicts the underlying principles. At each login, participants can choose one of 80 different themes to discover new knowledge and methods. A theme consists of tests to explore current habits, texts with health information and exercises to learn behavior change techniques. At the end of each theme, participants ask a question to themselves on how to act on the content. When returning, they evaluate their activities in daily life and proceed to discover another theme. Users can refine earlier questions or ask new ones, in order to stimulate increased knowledge and self-reflection as they proceed through the tool. **c** shows the exposure-response relationship. Participants attended quarterly visits during the first year. Exposure to the tool (number of completed themes) and the corresponding change of HbA1c relative to previous visit for each quarterly period during the first year was determined in all participants who used the tool (*n* = 205). A total of 161 participants used the tool as recommended (biweekly) during at least one quarter. Mean values are shown across all individuals for quarterly periods in which no themes (*n* = 114 periods), 1–4 themes (*n* = 228) and at least 5 themes (*n* = 311), respectively, were completed. Error bars are s.e.m.

Before constructing the digital treatment tool, we analyzed how psychological factors associate with the progression of glucose control in 195 individuals from the All New Diabetics In Scania (ANDIS) cohort, who attended semiannual visits over four years for analysis of HbA1c and completion of questionnaires (Fig. 1a; Supplementary Table 1). Increased perceived competence of diabetes and larger influence of life view on health-related habits were both associated with improved HbA1c over time (Supplementary Table 2). Of these patients, 22 also participated in interviews to examine needs, attitudes and barriers to diabetes self-management (Supplementary Table 3). The questionnaires and interviews taken together suggested a need to promote patient autonomy, connect self-management with overall life context and to extend focus on health-related areas beyond diet and exercise.

To address these unmet needs, we developed a treatment on the theoretical foundation of self-affirmation and implemented it as a digital tool to enable broad applicability^15^. This involved repeated testing by patients, including feedback on content, language, and relevance. The tool is composed of 80 themes on different areas (Supplementary Fig. 1, Supplementary Tables 4, 5). Large emphasis is placed on self-reflection to raise awareness of current priorities and explore different options for sustainable lifestyle changes (Fig. 1b). Self-reflection may support autonomy and has played an important role in, for example, later forms of cognitive behavioral therapy^16,17^. It has, however, not been of major focus in lifestyle management.

### Existence of effect in the randomization phase

We aimed to evaluate the tool in a setting that was as similar as possible to ordinary clinical conditions by observing the usage pattern and metabolic outcomes in patients with type 2 diabetes who had access to the tool in addition to usual care. Of the 667 individuals who underwent screening, 370 were enrolled. First, we assessed the existence of effect by randomizing the participants to access the tool immediately (*n* = 184; 26 lost to follow-up after initial visit) or wait for 12 weeks (*n* = 186; 8 lost to follow-up; Table 1, Supplementary Fig. 2, Supplementary Table 6).

**Table 1:** Demographic and baseline characteristics of participants in evaluation study^a^.

Characteristic	Tool (*n* = 184)	Usual care (*n* = 186)	All (*n* = 370)
Male sex – no. (%)	117 (63.6)	112 (60.2)	229 (61.9)
Age – years	63.6 (9.6)	63.0 (9.8)	63.3 (9.7)
Diabetes duration—years	4.2 (1.4)	4.1 (1.4)	4.2 (1.4)
Body mass index^b^	31.0 (5.3)	31.2 (5.1)	31.1 (5.2)
Glycated hemoglobin level—mmol/mol	63.6 (10.7)	62.9 (9.7)	63.2 (10.2)
Glucose-lowering medication—no. (%)			
None	7 (4.0)	7 (3.9)	14 (3.9)
Oral only	117 (66.5)	121 (66.1)	238 (66.1)
Oral and insulin	39 (22.2)	40 (21.9)	79 (21.9)
Insulin only	13 (7.4)	15 (8.2)	29 (8.1)
Socioeconomic status—no. (%)			
Employed	67 (43.2)	78 (45.9)	146 (44.8)
Unemployed	5 (3.2)	2 (1.2)	7 (2.1)
Retired	77 (49.7)	79 (46.5)	156 (47.9)
Sick−leave >3 months	6 (3.9)	8 (4.7)	14 (4.3)
Taking care of own household	0 (0)	3 (1.8)	3 (0.9)
Highest education—no^c^. (%)			
Basic level	26 (17.6)	25 (15.2)	51 (16.2)
Medium level	47 (31.8)	52 (31.5)	99 (31.5)
College/University	75 (50.7)	88 (53.4)	164 (52.2)

^a^Data are *n* (%) or mean (SD). Percentages may not total 100 because of rounding. Data on glucose-lowering medication, socioeconomic status and education were not available from all.

^b^The body-mass index is the weight in kilograms divided by the square of the height in meters.

^c^Basic level refers to up to nine years of education; medium level is up 12 years of education.

The average HbA1c decreased by 3.2 mmol/mol from baseline to twelve weeks (95% CI −4.5 to −1.9; *n* = 158) in participants with access to the tool. In participants randomized to wait, average HbA1c decreased by 1.3 mmol/mol (95% CI −2.6 to 0.0; *n* = 178). The mean difference between the groups was −1.9 mmol/mol (95% CI −3.7 to −0.1; *P* = 0.049). When adjusting for potential confounders related to study discontinuation, the mean difference was −2.2 mmol/mol (95% CI −4.1 to −0.3).

### Metabolic characterization of MOD and pronounced effects during randomization

Lifestyle changes have been shown to affect insulin resistance^18^ and lead to larger glycemic improvement in patients with well-preserved insulin secretion^19^, i.e. the typical characteristics of the recently identified MOD cluster^14^.

We investigated whether patients in the MOD cluster responded differently to the tool compared with patients in other clusters. Interestingly, participants with MOD characteristics (30%; Supplementary Table 7) randomized to use the tool had a pronounced reduction of HbA1c after twelve weeks compared to MOD participants on wait. The mean difference was −8.4 mmol/mol (95% CI −13.5 to −3.3). In contrast, there was no significant difference between randomization groups in participants without MOD (mean difference 1.1 mmol/mol [95% CI −2.5 to 4.6]). When evaluated as an interaction term in the statistical model, we observed a significant interaction between randomization group and MOD/non-MOD characteristics (*P* = 0.0067; *P* = 0.030 when adjusting for differences in age and initial HbA1c). This suggests that the response to the tool was larger in participants with MOD compared to those without MOD.

### Effect of the treatment from baseline to end of follow-up

In order to assess more long-term outcomes, the randomization groups were merged after the initial twelve-week period to enable all patients to use the tool during an extended open-label period (Supplementary Fig. 2). A total of 54 participants (of whom 26 were initially assigned to the tool and 28 to wait) did not provide any data beyond baseline (Supplementary Fig. 2, Supplementary Table 8), and 42 individuals were excluded from analysis because of changed glucose-lowering medication immediately after baseline measurements.

Study participants used the tool at their preferred pace but were recommended to complete a theme at least biweekly (i.e. every two weeks), in order to be exposed to the different areas covered by the tool while providing sufficient time to implement changes between sessions. In the 274 participants included in the long-term assessment (Supplementary Table 9), there was a correlation between exposure to the tool (number of completed themes) and the magnitude of metabolic improvement from baseline to end of follow-up (730 days on average [interquartile range 430–1021]). A relationship between exposure and HbA1c response was observed even at higher time resolution (three-month intervals), independent of overall usage (Fig. 1c).

**Fig. 2:**
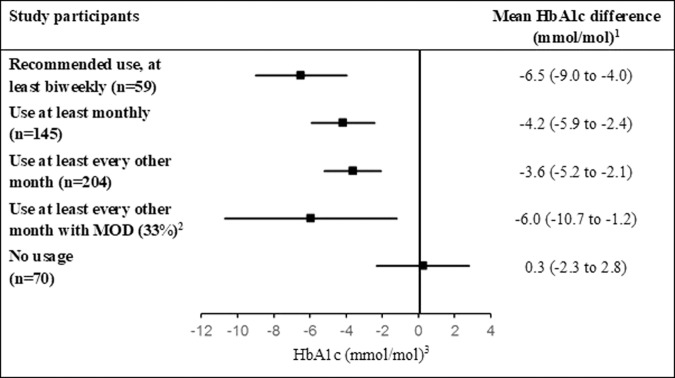
Change of HbA1c from baseline to end of follow-up in study participants at different usage patterns compared with controls. ^1^Estimated differences of study participants minus matched controls are presented as means (95% CI). ^2^Change of HbA1c when the tool was used at least every other month in participants with MOD characteristics compared with matched controls with MOD. A total of 32%, 28%, 33% and 21% of participants using the tool at least biweekly, monthly, bimonthly, and non-users, respectively, had MOD. ^3^Plots of mean HbA1c difference between participants and controls with 95% confidence intervals.

The primary analysis of participants using the tool at least biweekly (*n* = 59) demonstrated a 6.0 mmol/mol average reduction of HbA1c at the end of follow-up relative to baseline (95% CI, −8.5 to −3.5; 95% CI, −10.7 to −1.9 when adjusting for differences in baseline variables related to adherence and 95% CI, −8.7 to −3.5 when adjusting for differences in baseline physical activity; Supplementary Table 10). The matched controls followed over a similar time period (1:2 ratio; Supplementary Tables 11, 12) had an increase of 0.5 mmol/mol (95% CI, −0.5 to 1.4). The mean difference between the groups was −6.5 mmol/mol (95% CI, −9.0 to −4.0; *P* < 0.001; Fig. 2). HbA1c decreased also at less frequent exposure, and the mean difference compared with controls during total follow-up was 4.2 mmol/mol (95% CI −5.9 to −2.4; *n* = 145) when the tool was used at least monthly and 3.6 mmol/mol (95% CI −5.2 to −2.1; *n* = 204) when used at least bimonthly (every two months) (Table 2).

**Table 2:** Key secondary endpoints^a^.

Endpoint	Mean difference (95% CI)^b^		
	At least biweekly usage (*n* = 59)	At least monthly (*n* = 145)	At least bimonthly (*n* = 204)
Glycated hemoglobin level – mmol/mol	−6.5 (−9.0 to −4.0)	−4.2 (−5.9 to −2.4)	−3.6 (−5.2 to −2.1)
HOMA2−IR	−0.5 (−0.7 to −0.2)	−0.4 (−0.6 to −0.2)	−0.3 (−0.5 to −0.1)
HOMA2−B—%	20.4 (11.6 to 29.3)	15.4 (9.3 to 21.5)	17.4 (11.9 to 23.0)
Body weight—kg	−2.9 (−4.8 to −0.9)	−2.2 (−3.8 to −0.5)	−1.6 (−4.3 to 1.1)
Fat mass—%	−3.5 (−5.1 to −1.8)	−3.0 (−4.6 to −1.6)	−3.1 (−4.5 to −1.8)
Muscle mass—%	3.4 (1.8 to 4.9)	3.1 (2.0 to 4.3)	3.1 (2.1 to 4.1)
Fasting plasma glucose—mmol/l	−1.0 (−1.8 to −0.3)	−0.6 (−1.1 to −0.1)	−0.6 (−1.1 to −0.1)
Total cholesterol—mmol/l	−0.1 (−0.5 to 0.3)	−0.1 (−0.5 to 0.3)	−0.1 (−0.5 to 0.2)
LDL cholesterol—mmol/l	−0.1 (−0.5 to 0.2)	−0.07 (−0.4 to 0.2)	−0.1 (−0.4 to 0.2)
HDL cholesterol—mmol/l	0.1 (0.03 to 0.2)	0.1 (0.01 to 0.2)	0.1 (0.01 to 0.2)
Triglycerides—mmol/l	−0.01 (−0.4 to 0.3)	−0.04 (−0.4 to 0.3)	−0.03 (−0.4 to 0.3)
Systolic blood pressure—mm Hg	−11 (−18 to −5)	−12 (−19 to −5)	−12 (−19 to −6)
Diastolic blood pressure—mm Hg	−1 (−5 to 3)	−1 (−5 to 3)	−2 (−5 to 2)

^a^Changes from baseline to end of follow−up (average 730 days) in participants using the tool compared with controls on usual care.

^b^Estimated differences of study participants minus matched controls are presented as means with 95% CI at different usage patterns. Biweekly usage refers to usage every other week over at least a one−year time frame during follow-up.

To investigate the likelihood that the observed changes in users of the tool represented only naturally occurring fluctuations in glucose control that would be observed by chance in any cohort of diabetic patients, we analyzed the pattern of HbA1c progression in 13,561 patients with type 2 diabetes in ANDIS during three-year frames. Of the 1358 patients in ANDIS who had a baseline HbA1c of at least 52 mmol/mol and no known medication changes during the selected period, 16% exhibited a continuous reduction of HbA1c. In contrast, sustained HbA1c improvement was observed in 50% of study participants when the tool was used biweekly, in 39% when it was used at least monthly and 39% when used at least bimonthly. The relative number of patients with sustained HbA1c improvement was significantly higher in those exposed to the tool than what would be expected by chance (*P* < 0.001 for comparisons between ANDIS and study participants using Fisher’s exact test; Supplementary Table 13).

### Pronounced long-term effect in participants with MOD even at less-frequent usage

To analyze how the individual pathophysiology influenced the long-term response, we compared participants with or without MOD characteristics during follow-up. Participants with MOD (30%; relative numbers were unrelated to usage pattern) had a considerably larger HbA1c response, also when the tool was used less than recommended. The mean difference between the less-frequent users with MOD and matched controls with MOD was −6.4 mmol/mol (95% CI −11.0 to −1.8). When analyzed in a linear model, there was a significant interaction (*P* = 0.014) between MOD/non-MOD characteristics and users/controls. This shows that participants with MOD have a pronounced HbA1c response during long-term follow-up. Compared to non-MOD participants they had also larger improvement in insulin resistance (HOMA2-IR; mean difference −0.6 [95% CI −1.1 to −0.1]) and ameliorated insulin secretion (HOMA2-B; 16.8 [95% CI 6.5 to 26.9]), while the average change of body weight was −1.5 kg (95% CI −3.2 to 0.1; non-significant).

### Pathophysiological factors mediating improved glycemic control

The reduction of HbA1c was accompanied by decreased body weight, fat mass, insulin resistance and fasting glucose, increased muscle mass, reduced systolic blood pressure and improved HDL cholesterol, without observed changes in total cholesterol, LDL cholesterol, triglycerides, or diastolic blood pressure (Table 2). The changes of biological variables were accompanied by increased physical activity in study participants (758 kcal [95% CI 203 to 1312]; *n* = 71), as assessed by repeated International Physical Activity Questionnaires.

To better understand the pathophysiological changes in participants exposed to the tool, we examined to which extent reduced body weight and insulin resistance mediated the improved glycemic control. Reduced body weight was associated with improved HbA1c, and weight reduction was indicated to be a partial mediator of the total effect of using the tool (number of completed themes) on HbA1c improvement (Supplementary Table 14). Furthermore, improved insulin resistance was also associated with lower HbA1c and was indicated to mediate part of the effect of using the tool on HbA1c reduction (Supplementary Table 14). There was also an association between baseline BMI and improved HbA1c in users of the tool (beta = −0.23 [95% CI −0.44 to −0.03], stratified for sex; *n* = 204), and a 2.5 mmol/mol (95% CI −4.7 to −0.3) larger response in participants with BMI above median (30 kg/m^2^) compared to those below. Lower body weight and insulin resistance would be expected to ameliorate beta-cell stress and insulin secretory capacity^20^ .Accordingly, we observed an association between exposure to the tool and improved insulin secretion, measured by HOMA2-B (beta = 0.30 [95% CI, 0.02 to 0.58]; *n* = 204).

### Effective elements of the tool

To investigate the effective elements of the tool, we analyzed the different behavior change techniques that were incorporated into the themes and identified 8 techniques that were overrepresented in themes completed by users who responded to the tool by improved HbA1c from baseline to one year (Supplementary Table 15). These relate to self-affirmation theory and also to different aspects of existential health that were included on the tool to help users explore how overall life context relates to current habits and disease coping^15,21^.

When developing the tool, we postulated that self-reflection would promote glucose control by supporting participants to see personal relevance in health information. At the end of each theme, participants therefore asked a question to themselves on how to implement the information in daily life. To better understand how these questions are linked to study outcomes, we performed a semantic analysis using machine learning. Specifically, we aimed to determine to which extent the questions were abstract/reflective (e.g. ‘How can I view exercise as something meaningful rather than a burden?’) or more concrete (e.g. ‘What can I do to reach 10,000 steps a day?’). A transformer-based language model was trained with questions from users of the tool who were not part of the evaluation study. The resulting model achieved a classification accuracy of 88.4% on the test set. Of the questions asked by study participants, 55.5% were classified as concrete and 44.5% as abstract. Interestingly, the number of abstract questions was associated with improved HbA1c, while there was no association between the number of concrete questions and change of HbA1c (Supplementary Table 16). A similar pattern was observed when specifically studying participants using the tool as recommended; those who responded with reduced HbA1c from baseline to one year had a higher proportion of abstract questions compared to non-responders (Supplementary Table 16), further supporting the value of the reflective element.

### Cost-effectiveness of the Tool

Finally, we analyzed the health economic consequences of the tool compared to current standard of care. Patients using the tool biweekly would be at reduced risk for diabetic complications, including stroke, heart failure, macroalbuminuria and background retinopathy because of improved risk factor control (Supplementary Fig. 3). The yearly cost of the tool was approximated to $7.5 per regular user, including technical and administrative expenses (Supplementary Tables 17–19). Over a 20-year modeling period, implementation of the tool was estimated to result in cost savings of $4,116 and 0.5 more quality-adjusted life years for every patient using the tool biweekly (Supplementary Table 20). It would be cost-saving also at less-frequent usage ($2,770 for at least monthly and $2,231 for at least bimonthly usage; non-users would not incur any costs nor cost-savings). The model is based on a number of assumptions (Supplementary Tables 17–19) and the results are only indicative. We therefore performed a sensitivity analysis with different time horizons and changes in key parameters of the model, which indicated that the tool would be cost-saving up to yearly operating expenses between $110 and $411 per patient ($369 in the base case scenario), as compared with the approximated actual cost of $7.5 (Supplementary Table 21).

## Discussion

This study demonstrated significantly improved metabolic control over two years in patients with type 2 diabetes using the digital tool at least bimonthly in addition to standard anti-diabetic treatment. The tool adds a new facet to lifestyle management by its emphasis on self-reflection. The value of the self-reflective element was supported by natural language processing, showing that participants who asked a high number of abstract (reflective) questions to themselves had improved HbA1c. This observation is also in accordance with self-affirmation theory, which was the conceptual foundation of the study^15^.

There was a positive correlation between exposure and improvement of HbA1c, which was accompanied by reduced body weight and insulin resistance, ameliorated insulin secretion and lower systolic blood pressure. The reduction of blood glucose is partially mediated by weight loss and improved insulin resistance. This is also compatible with the pathophysiological data in participants with MOD, who exhibited a pronounced glucose reduction even when using the tool less than recommended. There is considerable interest in personalizing treatment for metabolic diseases based on the pathophysiology, but practical feasibility has been unclear. The findings could have general implications by providing a means to identify those who are most likely to respond to lifestyle treatment based on the pathophysiology. As an option to cluster assignment, a BMI cut-off may also be used^22,23^.

While previous meta-analyses have shown declining effect of lifestyle programs after six months, the metabolic improvement in response to the tool was sustained during two years, making this one of the longest and most extensive investigations of digital lifestyle treatment to date^9–12^. The study has also a number of limitations. No adjustment was made for multiple comparisons, and the variables should therefore be interpreted based on the overall pattern of outcomes. A fundamental problem to lifestyle studies is expectancy artefacts, as individuals randomized to wait may be affected in unpredictable ways, including changed motivation^24^. We therefore used two different control conditions. To better control for potential selection bias, study participants were first randomized to a wait list or to access the tool, with full allocation concealment. This was complemented by comparisons between study participants and matched controls in clinical registries, which increases the risk for selection bias but on the other hand reduces expectancy artefacts^24^.

Study participants may be more motivated to lifestyle changes than patients in general because of potential selection bias. The exposure-response relationship at three-month time resolution showed that change of HbA1c during different quarterly periods even within the same individual is related to usage (Fig. 1c). This suggests that the observed effects are not merely consequences of baseline traits or higher initial motivation in some participants but associated with exposure to the tool. It is also of interest to note that the glycemic response was larger in participants with a high proportion of abstract versus concrete questions, also when the tool was used as often as biweekly, indicating that the metabolic improvement is coupled with exposure to the reflective elements of the tool. To further examine to what extent the observed effects reflect spontaneous variations in glucose control, we investigated patterns of HbA1c progression, which demonstrated sustained improvement of HbA1c in a considerably larger portion of patients exposed to the tool than in patients on usual care in ANDIS.

Participants using the tool represented a wide range of age, education, and socioeconomic status. To correct for potential confounders in baseline variables related to adherence we applied analysis weights based on propensity scores. The potential influence of placebo effect related to study visits was also assessed. We observed that the annual increase of HbA1c in matched controls, who did not interact with study personnel, was similar to participants who attended visits but did not use the tool and also to the 195 individuals with prospective psychological assessments (Supplementary Table 1). Moreover, the quarterly exposure-response relationship is independent of placebo effects arising from study participation (Fig. 1c). This shows that even if a baseline placebo effect is present, usage of the tool has a significant effect on HbA1c. In light of the present data, we find it unlikely that placebo effects or confounders explain a major fraction of the observed effects in response to the tool.

It is of note that more participants discontinued in the intervention group (*n* = 26; 14%) than in the control group (*n* = 8; 4%) during the randomization period, probably because using the tool requires an active engagement that may conflict with other activities or interests to a higher extent than being on a wait list. The development of the tool involved semi-structured interviews on patient needs and preferences as well as repeated usability testing and iterative improvements to facilitate usability and uptake. A limitation is that we did not include any patient-reported usability measures during the subsequent evaluation study. Such measures could have provided information on how to further improve the relevance of the tool and given more detailed data on reasons for discontinuation, in addition to the metabolic outcomes.

Dropout rates could potentially have been reduced by enhanced efforts from study personnel to motivate participants. In order for digital lifestyle solutions to be scalable and broadly applicable, it is however essential that efficacy does not depend on in-person reinforcement or increased healthcare activities^9,13,25^. Thus, we purposely provided the tool as a stand-alone support under conditions that were as similar as possible to the everyday contexts of the patients. While this may reduce adherence compared with stricter regimes, it increases the general significance of the results by demonstrating what can be expected in real-life situations over extended time. The influence of different clinical circumstances on uptake, including integration of the tool with motivational activities by healthcare professionals, will be of interest to investigate in subsequent studies. Overall, approximately 15% discontinued during the initial twelve weeks, and 70 participants (25%) did not use the tool during the long-term follow-up. These rates are comparable with what has previously been observed in meta-analyses of digital tools^26,27^. We found that the tool is clinically valuable also when adherence is variable, suggesting broad clinical applicability. In view of the severe clinical need for scalable solutions to treat type 2 diabetes the findings offer a means for affordable lifestyle management that could benefit a large number of patients. During follow-up, average HbA1c was improved by approximately 6 mmol/mol both in the ~20% who used it biweekly and in the 30% of patients who have MOD characteristics, even at less-frequent usage. The HbA1c reduction is of similar magnitude to common glucose-lowering drugs, for example sodium glucose cotransporter type-2 (SGLT2)^28^ inhibitors or dipeptidyl peptidase-4 (DPP-4)^29^ inhibitors added to metformin or increased insulin treatment by 0.2–0.3 IU per kg^30^. The marginal cost is, however, fundamentally lower than pharmacological treatment and existing lifestyle intervention programs, and overall cost-effectiveness is consequently much less sensitive to variation in compliance. We demonstrate that even when not all patients use the tool as recommended it would still be cost-saving, in contrast to the cost-benefit model of most drugs and existing lifestyle support. A clinician could therefore provide the tool to all patients with type 2 diabetes, as any additional patient incurs a minimal cost, or direct it to patients with high body weight and insulin resistance, analogous to a tailored drug.

In summary, the study presents a self-reflective approach to lifestyle treatment that has previously been lacking. Patients with type 2 diabetes regularly using the tool had improved metabolic control over two years, with a pronounced response in a pathophysiological subgroup of obese insulin-resistant individuals. The tool does not require additional healthcare resources, which is often a major limitation for broad utility. It therefore opens an avenue to address the large unmet need for scalable treatment of lifestyle diseases with sustained efficacy.

## Methods

### Overall study description

We developed a lifestyle treatment on the theoretical foundation of self-affirmation and implemented it as a digital tool to enable broad applicability. The tool is web-based and is used as a stand-alone continuous support without requirement of additional healthcare activities. It is available in English and Swedish and is provided for free via academic institutions.

We conducted a clinical study (approved by the regional ethics review committee in Gothenburg; 651/2016) to test the hypothesis that glucose control would improve in patients with type 2 diabetes using the digital tool. The study aimed to investigate the pattern of usage and metabolic outcomes under conditions that were as similar as possible to real-life settings (ClinicalTrials.gov identifier NCT04691973, where the mixed design of the study including the randomized and observational portions are described in the Detailed description as of 31 Dec 2020). At study start, participants were randomized to access the tool or wait for twelve weeks (1:1 ratio). The two groups were then merged to enable all participants to use the tool during an extended open-label period for up to three years.

Participants used the tool at their preferred pace but received written recommendations within the tool to complete a theme biweekly. Each exposure to the tool takes approximately 10–30 min and typically involves one completed theme. Study personnel were instructed to remain neutral at blood sampling visits and not reinforce usage in order to assess the frequency of use and resultant outcomes that can be expected in real-life situations over extended time without the need for increased healthcare support.

The effect on HbA1c in participants using the tool was compared with both randomized controls and matched controls on usual care. Usual care refers to routine diabetes management and treatment based on the general guidelines from the European Association for the Study of Diabetes and the American Diabetes Association^1^.

The study was performed according to the Declaration of Helsinki and Good Clinical Practice. The protocol (Supplementary Data 1) was approved by the regional Ethics Review committee in Gothenburg and is appended with the paper. All participants provided written informed consent.

### Enrollment of participants and study procedures

Patients older than 35 years with prior documentation of type 2 diabetes and HbA1c ≥52 mmol/mol (standard glycemic treatment target according to current guidelines^1^) were eligible for enrollment. Diabetes mellitus was diagnosed in routine healthcare based on the WHO criteria (fasting plasma glucose ≥7.0 mmol/l or 2-h post-load plasma glucose ≥11.1 mmol/l or HbA1C ≥48 mmol/mol). All participants provided written informed consent. Exclusion criteria were: type 1 diabetes, maturity-onset diabetes of the young (MODY), secondary diabetes, other conditions, treatments or participation in clinical studies that in the judgment of the investigator could affect the evaluation, or conflicts of interest (such as associations with the study team, funders, authorities, universities or other public or private bodies).

Participants were recruited via letters sent to patients with a known history of type 2 diabetes in the ANDIS registry or by advertisements. Individuals who were interested in taking part contacted the study personnel via telephone, regular post, email or through a dedicated web page. Those who met the study criteria at the first visit were included and invited to subsequent visits every third months during the first year and thereafter every six months over a total follow-up period of up to three years. Each visit lasted approximately 20 min and included blood sampling and measurements of length (first visit only), body weight, blood pressure and bioimpedance. Participants were also requested to report medication changes. Total follow-up time varied between participants, as they were not all recruited simultaneously.

The study center was located at Scania University Hospital, Malmö, Sweden. Participants received travel reimbursement to enable individuals from all over the region (southern Sweden), also outside the main cities, to take part. No other financial incentives were provided. Study participants were managed by their ordinary healthcare providers throughout the study.

Healthcare providers and study personnel were not involved in the participants’ activities on the tool. The participants used it at their preferred pace but received written recommendations within the tool to complete a theme at least every other week. The recommended frequency of use intended to enable sufficient time to implement changes between sessions while maintaining awareness of the questions and being exposed to the different areas covered by the tool during a year. It was in line with the preferences expressed by patients in the test sessions and with previous recommendations for digital lifestyle tools^26^. Participants received regular emails to prompt continuous usage but no reinforcement by study personnel, who was instructed to remain neutral at visits, as we wanted to assess the natural usage pattern and resultant outcomes without external interference. Technical problems were referred to a study coordinator, who also responded to requests to clarify content in a general manner without providing personal advice.

### Randomization procedures

After the first visit, participants received an introductory email with a secure web link to a personal study account and instructions on how to get started. By clicking the link, they set a password, completed a questionnaire, and were randomized via a web-based system to have immediate access to the tool or wait for twelve weeks. In this manner, allocation was concealed to both participants and study personnel at the first visit (whether participants were eventually assigned to the tool or usual care was, however, open-label).

Participants randomized to wait received usual care via their ordinary healthcare provider. They were invited to a second visit after twelve weeks for blood sampling and physical examination. Following that visit, they received an email message with a link to complete the questionnaire once again and were then able to access the tool.

The randomization sequence (block size of eight, unknown to study personnel) was generated by external statisticians, who had no contact with participants or study personnel. It was uploaded by external technical staff to the web-based allocation system. Thus, the generation of the random sequence, participant enrollment by study personnel, and the web-based system for allocation to randomization groups were clearly separated.

### Selection of matched controls

After the initial 12-week period, all study participants had access to the tool and were compared with matched controls on usual care. Controls were selected from patients with type 2 diabetes in the ANDIS registry. ANDIS was approved by the regional ethics review committee in Lund (584/2006 and 676/2012) and aims to register all incident cases of diabetes in Scania, which is one of the largest regions in Sweden with 1,200,000 inhabitants in both rural and urban areas and a wide distribution of socioeconomic background. Approximately 23,000 diabetic patients (>90% of the estimated number of eligible cases in the region) are included as of December 2020. Most individuals with type 2 diabetes are managed in primary care. Fasting blood glucose, C-peptide, glutamic acid decarboxylase (GAD) antibodies, length and body weight are measured at registration. Prospective data on metabolic variables and medication are obtained via the Swedish National Diabetes Registry and clinical registries on drug prescription and laboratory tests.

The controls were matched exactly with study participants on gender and on Mahalanobis distance based on age (at the index date), body mass index (BMI) and HbA1c. The first possible index date for controls was set to be at least two years after the diagnosis date. The index date was selected at random among all registrations meeting the requirements for available follow-up time with no medication change during follow-up (as for the intervention group). Selection of the index date and potential controls were done in a Statistical Analysis System (SAS) program. Balance before and after matching was evaluated using the standardized mean difference (SMD).

### Subgroup analysis of patients with mild obesity-related diabetes

A data-driven cluster analysis of 9000 patients with diabetes has recently been performed in the ANDIS cohort based on six variables measured at diagnosis: GAD antibodies, age, BMI, HbA1c, and homeostasis model assessment-2 estimates of insulin resistance (HOMA2-IR) and beta-cell function (HOMA2-B)^14^. Four clusters of type 2 diabetes patients were highlighted, each with different pathophysiological characteristics (a separate cluster corresponding to autoimmune diabetes was also identified). The findings have been replicated in additional cohorts^14,31^. The clusters do not represent separate disease entities^22,23,32^ but may be used as a practical means to distinguish individuals with different pathophysiology. Cluster assignment of individual patients can be done clinically via an online program that uses age at diagnosis, sex, BMI, HbA1c, fasting glucose and C-peptide as input variables^14^ .

One of the clusters, termed Mild Obesity-related Diabetes (MOD; not to be confused with MODY), is characterized by high BMI and insulin resistance but relatively well-preserved insulin secretion. Studies on diabetes prevention have indicated that the response to lifestyle interventions may be larger in those with high BMI and insulin resistance, but data are conflicting^12,13^. Moreover, it is currently unclear how the individual pathophysiology influences the response to lifestyle interventions in individuals with manifest type 2 diabetes. Since lifestyle changes have been shown to affect insulin resistance^18^ and lead to larger glycemic improvement in patients with relatively well-preserved insulin secretion^19^ (i.e. the typical features of MOD), we hypothesized that the tool would be more effective in patients with MOD than in patients without MOD. Study participants and matched controls were therefore categorized as MOD and non-MOD, respectively, based on the published clustering methodology^14^. Comparisons were adjusted for differences in baseline HbA1c between MOD and non-MOD participants. The variation in BMI between MOD and non-MOD corresponds to the typical phenotypes, and adjusting for baseline BMI would therefore blur the clinical features that we aim to contrast.

### Baseline data

The following baseline data were collected at the first visit: age, sex, BMI, level of education, socioeconomic status, smoking habits, time since diabetes diagnosis, and current glucose-lowering treatment.

### Clinical study outcomes

The primary study variable was HbA1c in blood.

Secondary variables included: body weight, blood pressure, fat mass and muscle mass as estimated by bioimpedance (using a BIA101 from Akern, Pisa, Italy to obtain values of resistance and reactance), fasting blood glucose, total plasma cholesterol, plasma low density lipoprotein (LDL) cholesterol, plasma high density lipoprotein (HDL) cholesterol, plasma triglycerides, and HOMA2-IR and HOMA2-B based on fasting glucose and C-peptide.

Blood samples were taken in the morning (between 7.30 and 10.00). Participants were instructed to be fasted since 10 pm the previous day and to avoid nicotine use the same day and alcohol consumption and strenuous physical activity within 24 h of the visit. Fasting blood glucose was measured at the study center using a HemoCue Glucose System (HemoCue AB, Sweden). All other blood analyses were performed at the central hospital laboratory (Malmö, Sweden). HbA1c was analyzed according to International Federation of Clinical Chemistry (IFCC) standard by a Capillary 3 TERA Hemoglobin A1c Kit. C-peptide, total cholesterol, LDL cholesterol, HDL cholesterol and triglycerides were measured on Cobas (Roche Diagnostics, Mannheim, Germany).

HOMA2-IR and HOMA2-B were calculated based on C-peptide concentrations (which performs better than insulin in individuals with type-2 diabetes) using the HOMA calculator (University of Oxford, Oxford, UK)^33^.

Blood pressure was measured using a standardized cuff adapted to the size of the participant’s arm after the participant had rested in a sitting position for at least ten minutes. Height was recorded in centimeters and body weight in kilograms, to one decimal place, with light clothing and no shoes.

Bioimpedance measurements were done using a BIA101 to obtain values of resistance and reactance. Fat-free mass (FFM) was calculated using the Geneva single BIA equation (equations 1 and 2):

1 $$\begin{array}{l}{\mathrm{FFMmale}}=-{4.104} \,+\, ({0.518} \,\times\, {\mathrm{height}} \,\times\, {\mathrm{height}} /{\mathrm{resistance}}) \,+\, ({0.231} \,\times\, {\mathrm{weight}})\\\qquad\qquad\quad\; \,+\, ({0.130} \,\times\, {\mathrm{reactance}}) \,+\, {4.229}\end{array}$$

2 $$\begin{array}{l}{\mathrm{FFMfemale}}=-{4.104} \,+\, ({0.518} \,\times\, {\mathrm{height}} \,\times\, {\mathrm{height}} /{\mathrm{resistance}}) \,+\,\\\qquad\qquad\qquad\;\; ({0.231} \,\times\, {\mathrm{weight}}) \,+\, ({0.130} \,\times\, {\mathrm{reactance}})\end{array}$$

Total body water (TBW) was estimated using the multi-component sex-specific model developed by Sun and colleagues (equations 3 and 4):^34^

3 $${\mathrm{TBWmale}} = {1.20} \,+\, ({0.45} \,\times\, {\mathrm{height}} \,\times\, {\mathrm{height}} / {\mathrm{resistance}}) \,+\, ({0.18} \,\times\, {\mathrm{body}} \,{\mathrm{weight}})$$

4 $${\mathrm{TBWfemale}} = {3.75} \,+\, ({0.45} \,\times\, {\mathrm{height}} \,\times\, {\mathrm{height}} / {\mathrm{resistance}}) \,+\, ({0.11} \,\times\, {\mathrm{body}} \,{\mathrm{weight}})$$

Fat mass (FM) was calculated by deducting the FFM from the total body weight of the individuals. Muscle mass (MM) was estimated based on the assumption that MM is the remaining mass of the FFM after deducting extracellular water (~29%) and bone mineral (~7%)^35^ .

### Patient-reported outcomes

Every three months, the participants completed a questionnaire with the European Quality of-Life (Euro-Qol) Five Dimension Five Level Scale (EQ-5D-5L), a preference-based measure of quality-of-life. The questionnaire was separate from the tool and completed by participants via their study account online using a quarterly reminder system. The responses from EQ-5D-5L were converted to a sum score using the official scoring system provided by Euro-Qol^36^. Participants were instructed to complete the questionnaire on their own to avoid observer bias. EQ-5D-5L score ranges from 0 to 1 with higher score indicating better quality-of-life. EQ-5D-5L scores were not available from controls, and change of EQ-5D-5L score was therefore analyzed from baseline to one year in study participants using paired comparisons.

Participants also completed the International Physical Activity Questionnaire (IPAQ), which assesses intense and moderate physical activity as well as walking during the last seven days^37^ .This questionnaire was completed by users within the tool at less strict time intervals than EQ-5D-5L. IPAQ was therefore included only as an exploratory variable (it was completed at least twice by a total of 71 participants). Responses were converted to Metabolic Equivalent Task minutes (MET-minutes) per week according to the IPAQ scoring protocol^37^. MET-minute scores are equivalent to kilocalories for a 60-kg person, and the number of kilocalories were computed from MET-minutes as

MET-minutes x (body weight in kilograms/60 kilograms).

Supplementary Table 9 shows data on baseline IPAQ assessments with no indication of systematic differences in initial physical activity related to subsequent frequency of using the tool.

### Adjustment for potential confounders

A fundamental problem in lifestyle interventions is control comparisons. The expectancy artefacts that may occur when individuals are randomly assigned to the least preferred option, whether it is a wait list or strict continuation of usual care, are difficult to predict. It could be envisioned that assignment to a wait list (with promise of the tool after 12 weeks) for some individuals is more motivating than being assigned to mere usual care (never access to the tool within the study) and could inspire healthy behaviors already during the wait period, while others may of course react in opposite ways. We also reasoned that the risk for discontinuation is higher when participants are randomized to strict usual care compared with wait list controls.

Based on this reasoning we used two different control conditions: wait list controls during a randomization period, with the inherent risks of expectancy artefacts, and an extended open-label period where all participants had access to the tool and were compared with matched controls on usual care, which increases the risk for confounding effects but on the other hand reduces expectancy artefacts. We performed a number of analyses to adjust for potential confounders related to the different control conditions.

First, in the randomization phase, we used analysis weights based on propensity scores to adjust for potential confounders between participant randomized to wait and to access the tool as a result of participant drop-out during the randomization phase. The weights are based on the probability of continuing the study and are applied to statistically adjust the composition of the group of non-dropouts who accessed the tool, taking the baseline characteristics of the drop-outs into account.

Second, in the randomization phase, a total of 99 of the 143 participants randomized to access the tool used it as recommended (at least biweekly) during the twelve weeks. To adjust for any systematic differences related to adherence, we applied analysis weights based on the probability of using the tool as recommended to statistically adjust the composition of the groups in order to estimate the mean difference if the groups were more comparable in terms of baseline characteristics.

Third, in the subsequent long-term assessment period, we also used propensity scores to account for potential confounders related to frequency of using the tool. Specifically, we contrasted biweekly users with non-users in order to estimate the effects that would be observed if the frequent users had the same composition as non-users in terms of sex, age, BMI, diabetes treatment, socioeconomic status, education and disease duration.

Analysis weights based on propensity scores is a state-of-the-art method to adjust for potential confounders, and the comparisons were made using a linear model with change in HbA1c as the dependent variable and participant group as the only independent variable. We used both unadjusted and adjusted analyses, where the adjustment is intended to correct for confounders related to adherence or dropout between participant groups. The analysis weights were defined as follows for the different analyses:

First, the inverse of the probability of being a non-dropout in the treatment group and one in the wait group during randomization; second, the inverse of the probability of using the tool as recommended in the access group and one in the wait group during randomization; third, the inverse of the probability of being a user of the tool for the group actively using it and the inverse of one minus the probability of being a user of the tool for the group of non-users.

The probabilities were estimated using gradient boosting method (GBM) with shrinkage 0.001, interaction depth 3 and the number of trees optimized to minimize the average standardized mean difference using cross validation. The weighed analyses used a robust sandwich estimator for the standard error to account for the weights. Missing data is handled by the GBM as the algorithm splints only on observed values. The analysis was done using R 4.0.2 and specifically the twang package for the estimation of the analysis weights.

In addition to potential confounders captured by the measured baseline variables, differences in other baseline traits or initial motivation may also confound the results. It is very difficult to completely rule out all possible confounders in lifestyle studies, but we performed a range of analyses to address this matter as thoroughly as possible.

- We did a quarterly exposure-response analysis to assess whether change of HbA1c over time within the same individual is directly related to whether the tool is actively used or not during different quarters. This indicates whether the observed effects are consequences of baseline traits and higher initial motivation in some participants or coupled with exposure to the tool.
- Since the exposure-response analysis does not fully exclude the possibility that frequency of use could be a marker for fluctuating motivation during the follow-up period, we also performed a semantic analysis of user behavior on the tool (see below). Specifically, we examined whether the glycemic response differed based on the number of concrete and abstract questions, respectively, asked by participants. This analysis aimed to better understand whether the metabolic improvement is a result of exposure to specific elements of the tool.
- To further address to what extent the observed effects may reflect spontaneous fluctuations in glucose control, we analyzed patterns of HbA1c progression in the ANDIS cohort and compared these data with HbA1c progression in users of the tool (see below).

### Exposure-response analysis

Participants attended quarterly visits during the first year. We examined the number of completed themes and the corresponding change of HbA1c relative to previous visit for each quarterly period in all participants. The average change of HbA1c during quarterly periods with none completed themes, with 1–4 completed themes and with at least 5 completed themes across all participants was determined.

### Patterns of HbA1c progression

We analyzed trajectories of HbA1c progression in 13,561 patients diagnosed with type 2 diabetes between 2008 to 2018 in ANDIS with prospective HbA1c data measured in routine healthcare. A total of 4,602 patients had at least three HbA1c measurements over a three-year time frame at an average of 1,320 days (3.6 years) from diagnosis. Patients in ANDIS who were also included in the evaluation study of the tool were removed.

We identified four common patterns of HbA1c trajectories:

1. Sustained decrease relative to baseline: at least one HbA1c measurements was lower than baseline HbA1c at the start of the three-year time frame and all other measurements were lower than or equal to baseline.
2. Sustained increase relative to baseline: at least one HbA1c measurements was higher than baseline HbA1c and all other measurements were higher than or equal to baseline.
3. Oscillatory, predominantly decreasing: the majority, but not all, of the measurements were lower or equal to baseline HbA1c.
4. Oscillatory, predominantly increasing: half of more, but not all, of the measurements were higher than baseline HbA1c.

A total of 1358 patients had a baseline HbA1c of at least 52 mmol/mol and no known change of glucose-lowering medication during the three-year time frame (similar to the patients using the tool who were included in the long-term analyses). The average follow-up time between first and last HbA1c measurement for these patients was 632 days (as compared with 730 days for users of the tool).

We analyzed HbA1c progression in users during the follow-up period of the tool in a similar manner.

The number of patients corresponding to each pattern was compared between ANDIS patients and users of the tool using Fisher’s exact test.

### Analysis of behavior change techniques

To better understand the effective elements of the tool, we investigated, as an explorative analysis, whether specific behavior change techniques (BCT) were overrepresented in themes completed by users who responded to the tool by improved HbA1c from baseline to one year. These analyses focused on the first year of the study, during which participants attended quarterly visits.

For every quarterly period (i.e. the interval between two study visits), we examined which participants had completed a particular theme. We further analyzed how many of those participants had lower HbA1c at the end of the period than at the start of the period (“responders”) and how many had similar or higher HbA1c (“non-responders”). Next, the 2–4 BCT that were included in the theme were weighted according to their dominance (Supplementary Table 5). The number of responders and non-responders, respectively, were multiplied by this weight to obtain a responder and a non-responder score for each of the included BCT. These scores were summed for the four quarters.

The procedure was repeated for every theme, and the resultant responder and non-responder scores for each BCT were summed across all themes.

### Semantic analysis of questions

After each theme, the participants ask a question to themselves on how to implement the information in daily life. To better understand how these questions may influence study outcomes, we performed a semantic analysis. As we had postulated that self-reflection would promote glucose control, we specifically aimed to determine to which extent the questions were abstract (reflective) or more concrete.

The semantic analysis was performed using a machine-learning technique termed BERT (Bidirectional Encoder Representations from Transformers). BERT is a transformer-based language model trained with a cloze-task objective^38^ and is trained to fill in (artificially generated) blanks in natural language text. The resulting “pre-trained” model can then be re-trained (“fine-tuned”) on new tasks using only limited training data and small model modifications^38^.

In this study, we used a BERT model via the National Library of Sweden, called KB-BERT (Kungliga Biblioteket-BERT)^39^. KB-BERT is pre-trained on Swedish text from the 1940’s and onward, consisting of digitized newspapers, official reports of the Swedish government, legal deposits to the National Library, various social media data, and Swedish Wikipedia. KB-BERT is the most performant Swedish BERT model, outperforming multilingual BERT and other Swedish BERT models on a range of fine-tuning tasks^39^.

We used Huggingface transformers to retrieve the pre-trained KB-BERT model and trained it on classifying questions as either concrete or abstract using standard procedures for single-document classification. i.e. the classification output representation is fed to a single layer softmax classifier, training “all” parameters of the model.

KB-BERT was trained with questions from users of the tool, including test users, who were not part of the evaluation study. The total number of questions was ~5000, and the number of unique questions was ~1900. For evaluation purposes, we constructed a 50-50 train-test split over all unique questions (constructing a train-test split over the ~5000 questions would invalidate the test set, as data points in the test set are very likely to be present in the training set.) Thus, we trained the abstract-concrete classifier on half of the unique questions, and evaluated the resulting model on the remaining half. We trained the model for one epoch using an AdamW optimizer with a constant learning rate of 1E-5.

The resulting model achieved a classification accuracy of 88.4 % on the test set. Of the questions asked by study participants, 55.5% were classified as concrete and 44.5% as abstract, with a few examples as below:

Abstract questions:

- How limited am I and what opportunities do I have to influence my health?
- Am I prioritizing what is really meaningful?
- Could my family also benefit from me changing breakfast habits?
- How can I see moderation as something valuable rather than a sacrifice?

Concrete questions:

- What can I do to lose 5 kg of body weight?
- How can I reprioritize my daily life to get more time for exercise?
- What could I change to reduce the glycemic index of my breakfast?
- How could I eat less?

### Structure of the tool and psychological factors associated with glucose control

Before constructing the digital tool, we analyzed how psychological factors of importance to diabetes management associate with the progression of glucose control in individuals with type 2 diabetes. A total of 2000 individuals in the All New Diabetics In Scania (ANDIS) registry in Sweden were invited via letters, of which 195 accepted to attend semiannual visits over an average of 32 months (interquartile range [IQR], 24–42; Supplementary Table 1). All patients provided informed consent (approved by the regional ethics review committee in Lund, 2013/84).

At each visit (at Scania University hospital, Malmö, Sweden), the level of glycated hemoglobin (HbA1c) in blood was analyzed. The patients also completed a questionnaire including the following scales:

- Appraisal of Diabetes Scale^40^, which addresses the impact of diabetes on daily life. The response to the seven items on a 5-point Likert scale was summed and used for analysis. The smaller the total score, the more positive the appraisal strategy.
- Perceived Competence for Diabetes Scale;^41^ the average response to the four items on a 7-point Likert scale was used for analysis. A high score implies high self-rated capacity to manage diabetes.
- Treatment Self-Regulation Questionnaire^41^, which addresses motivation to lifestyle changes along two subscales: controlled motivation and autonomous regulation, reflecting extrinsic and intrinsic motivation, respectively. The average score for each subscale was used for analysis.
- Perceived influence of life view on health-related habits with a 10-point Likert scale^;^^42^ the score from 1 (lowest) to 10 addresses to what extent reflections on overall life context are influencing health-related habits.

The association between each score and HbA1c across visits was analyzed using a linear model in which values from each visit were included as discrete observations and grouped by study subject.

The unstandardized beta coefficients from the linear models are reported with 95% confidence intervals in Supplementary Table 2. Increased perceived competence of diabetes and increased influence of life view on health-related habits were both associated with improved HbA1c over time.

Of these participants, 30 were also invited via letters to take part in interviews to examine needs, attitudes and barriers to diabetes self-management (approved by the regional ethics review committee in Lund, 2014/702). We judged this number to be appropriate to meet the aims of the interviews, be practically manageable and in line with principles for good qualitative methodology. The interviewees were selected based on maximum variation sampling to ensure a diverse sample based on age, disease duration, treatment and HbA1c. The interviews were conducted by a psychologist with extensive knowledge of diabetes and diabetes research, who had no prior relationship with the participants.

### Analysis of interviews

The interviews were semi-structured and covered the experience of having diabetes, barriers to and previous experience of making lifestyle changes, perceived competence for diabetes management, as well as current self-management support and goals.

A full description of the interviews is presented elsewhere^43^. In brief, all interviews were audio recorded, transcribed verbatim and checked against audio recordings for accuracy. The qualitative data analysis software program ATLAS.ti (version 8.2.4) was used to organize the analysis process.

A total of 22 individuals accepted taking part in the interviews and provided written informed consent (Supplementary Table 3). Two participants had used physical activity applications via their mobile phone; the remainder had not previously been exposed to digital tools for health-related issues.

Participants reported a range of barriers to diabetes self-management, including stress, fatigue, pain, influence of habits, uncertainty about which health information to trust, and a sense that lifestyle changes proposed by healthcare was incompatible with current life situation. There was considerable variation in perceived urgency and distress related to diabetes self-management. Participants further reported limited inspiration from current lifestyle support in healthcare and difficulties implementing long-term changes.

The participants were also asked about personal needs and expectations of a digital lifestyle tool. They expressed a wish to use it as a source of encouragement and to get reliable information on diabetes management with a long-term perspective. Some also requested help with stress management and emotional support.

The questionnaires and interviews taken together suggested a need to promote patient autonomy, connect diabetes self-management with overall life context and help patients make decisions based on health information. This is also in agreement with previous reports from large-scale surveys of individuals with diabetes^44^. To address these unmet needs, we developed a digital tool on the theoretical foundations of self-affirmation and motivational interviewing.

Self-affirmation theory is based on the observation that people may react defensively when reminded of unhealthy behaviors and therefore reject information that threatens self-integrity, i.e. the concept of oneself as acting in line with existing norms and personal values and beliefs^15^ .The defensive reactions may include denial and avoidance in order to minimize the threat and preserve self-integrity. Self-affirmation theory postulates that perceived threats to one domain (e.g. sedentary behavior) can be managed more effectively by reflecting on strengths in other domains. From that broader perspective people may regard changes to specific behaviors as less threatening to overall self-integrity, leading to a less defensive attitude. Self-affirmation is associated with both greater intentions for behavioral change and actual behavioral change^21,45,46^.

Motivational interviewing (MI) has previously been demonstrated to affect dietary behavior and weight reduction^17,47^. We incorporated the principles of MI in the tool to promote reflection on ambivalence and commitment to change, which has been shown to increase intrinsic motivation^48^. To enable this without a traditional interviewer, the digital tool contains a large number of questions to stimulate self-reflection. Self-reflection may support autonomy^16^ and has played an important role in, for example, later forms of cognitive behavioral therapy^49^. It has, however, not been of major focus in diabetes self-management.

### Outline of the tool

The tool was developed in an iterative manner with repeated testing by patients, including feedback on usability and content. Patients who had participated in the interviews were exposed to computer-based assignments focusing on self-reflection and on viewing current lifestyle in a larger context, in line with self-affirmation theory and MI (approved by the regional ethics review committee in Lund 2015/563). Some participants found these assignments difficult and too personal, while the majority considered them helpful in order to think in new ways about type 2 diabetes and reflect on current priorities. Participants highlighted that the focus on self-reflection, which was regarded as rather unusual in diabetes care, could increase the sense of personal relevance and adherence to health information.

The tool was also demonstrated to physicians, nurses, dietitians and psychologists with extensive experience of meeting patients with lifestyle-related diseases. Patients and healthcare professionals tested early versions of the tool at individual sessions and commented on usability, language, design, perceived purpose, relevance and opportunities for improvement.

After each session they were asked to respond to the following questions: Would you personally have use for the activity you just did? How was it to do the activity? Can you provide three positive and three negative points from your experience? Is there something you think needs to be improved? What would you say was the purpose with this activity? Do you think the activity is of relevance to your diabetes disease? Was something in language or design unclear?

The testing and iterative modifications were conducted by psychologists and physicians in the study team and by external technical experts.

Based on the feedback and observations of user behavior, a complete digital tool was developed (in Drupal, version 7). The tool is web-based with a responsive design to enable patients to use it via their own computer, tablet or mobile phone. All data are transferred via secure protocols and stored on secure servers.

The tool is composed of 80 different themes, focusing on diet and exercise but also a range of other areas, including stress management, decision-making, social interactions, loneliness, and the emotional burden of having diabetes. Previous systematic reviews have highlighted the importance of including defined behavior change techniques (BCT) to better understand the effective elements of lifestyle interventions and generalize findings^5^. We therefore based the themes on the comprehensive theoretical framework and taxonomy proposed by Michie and colleagues^50^, which has been widely accepted^5^. Each theme of the tool incorporates 2–4 BCT (24 BCT are used altogether). Supplementary Table 4 describes the different BCT, and Supplementary Table 5 presents the full list of themes on the tool.

A theme takes approximately 10–30 min to complete and contains one or more of the following components:

- A self-assessment test with automated feedback, aiming to raise awareness of current behavior.
- An exercise such as evaluating short- and long-term benefits and losses associated with health-related goals, in order to identify willingness and barriers to change^3^. Other exercises may involve time prioritization, mindful eating, and methods to cope with distress and automatic thoughts.
- Informative texts (approximately 500–800 words per text) on topics of relevance to health and lifestyle-related diseases. Texts are based on international consensus recommendations for lifestyle management^1,51^. All texts were written by scientists specifically for this tool, underwent careful editing for style, tone and language by a panel of physicians, journalists and authors, followed by final approval for accuracy and clarity by the principal investigator.

The tool is accessed via any web browser using a study ID and a password. At each login, users select a theme they consider to be of relevance. At the end of the theme, they ask a question to themselves to promote reflection on how the information and insights from the theme could be implemented in daily life. This methodology is in line with self-affirmation theory and MI^15,16,48^ and the needs expressed in patient interviews. It aims to facilitate sustainable lifestyle changes by raising general awareness of current priorities and helping patients explore different options and choose their own course of action.

In every theme, users can choose from three to five example questions or write a question on their own. Questions typically take the form “How can I…”, for example:

- How can I reprioritize to get more time to cook healthy food?
- How can I stop blaming myself and focus on my good habits?
- How can I restructure my home environment to reduce the signals that make me eat so often?

They may also take a freer form, for example:

- Who could motivate me to change my lifestyle?
- Have I really taken in that diabetes is a serious disease and am I doing what I can to manage it?
- Am I eating now because I am hungry or because of old habits?
- What is the smallest thing I can do to get more physical activity into my everyday life?

After completing a theme and choosing a personal question, the users log out; they are encouraged to ponder on these questions in their daily lives and return to the tool within two weeks. Users are then given the opportunity to comment briefly in a diary on whether the question led to any behavioral changes. They select a new theme or revisit one they have previously found valuable. Users can refine earlier questions or ask new ones, in order to stimulate an evolution of questions as they proceed through the tool.

The various themes allow users to see how different areas are connected and how problems in one domain, e.g. unhealthy eating, could be managed by changes in other domains, such as stress coping. As the initial patient questionnaires and interviews identified a need to frame diabetes management within a larger perspective, we also include a set of themes covering the different aspects of existential health that the World Health Organization (WHO) has proposed^52,53^. These themes aim to stimulate questions on overall life context and how it relates to current habits and disease coping (Supplementary Table 5).

The tool is supposed to be used as a continuous support without a finite number of sessions (Supplementary Fig. 1). There is a diary function for bookmarking texts, writing comments and getting an overview of personal progression in terms of themes completed, questions asked and changes made in daily life. Users receive regular email prompts about their next round. There are brief (~1 min) summary videos after each theme and several instructional videos explaining the rationale of the tool. Specific functionalities for glucose monitoring, medication adherence or data tracking are not included, as such features are already addressed by several existing programs.

The tool is maintained and provided via academic institutions (Universities of Gothenburg and Lund, Sweden). It is technically prepared for multiple languages with a language select function and is currently available in English and Swedish. The tool is constructed to enable large scalability and can be implemented as a stand-alone support or combined with other lifestyle management activities. Providing the service to 50,000 users (including the current study participants and users taking part in an international health promotion program involving the tool) requires a part-time coordinator (who responds to technical issues, ~10 requests per week per 10,000 users) and technical personnel (50% full-time employee for updates and maintenance) in addition to server costs.

To maintain privacy, healthcare providers are not able to access user data. Technical functions are, however, prepared to enable users to download a report of their personal questions and reflections in case they wish to share all or parts of their activities on the tool with healthcare professionals to facilitate consultation (this functionality was not employed in the present evaluation study).

### The IHE Diabetes cohort model for cost-effectiveness analysis

We used a validated model, the Institute for Health Economics (IHE) Diabetes Cohort Model, to evaluate the cost-effectiveness of the tool and conducted multiple sensitivity analyses^54^. The analysis was based on assessment of risk factor control, which is a treatment goals because it has an impact on long-term risk of complications. Model simulations based on risk factors are often used in diabetes interventions, rather than within-trial analyses, as the time horizon of most trials is too short to cover expected benefits.

The IHE model uses cohort data to estimate the cost-effectiveness of treatment for diabetes^54^. It is constructed with Markov health states representing important microvascular complications (retinopathy, neuropathy and nephropathy) and macrovascular complications (myocardial infarction, ischemic heart disease, heart failure and stroke). The model is flexible with several input parameters, including baseline characteristics of the cohort, choice of risk equations, treatment sequences, unit costs and quality-adjusted life-year (QALY) weights. Model results (costs and QALYs) are discounted using a 3% annual discount rate.

### Base case scenario of cost-effectiveness analysis

The baseline characteristics used in the model (Supplementary Table 18) were based on mean values of study participants using the tool as recommended and matched controls (data from the ANDIS registry). Since microvascular complications and a few biomarkers included in the model were not reported in detail in the ANDIS registry, we extracted corresponding data from the UK Prospective Diabetes Study (UKPDS)^55,56^ or Swedish clinical registries^57–59^ for patients with similar age, disease duration and glycemic control, in order to obtain as precise measures as possible. The UKPDS study has established evidence for the importance of risk factor control in type 2 diabetes and is still considered some of the best-available data on HbA1c progression for standard of care treatment^55,56^ .

Since the model is based on a cycle length of one year, the change of relevant biomarkers from baseline to one year in participants using the tool as recommended and matched controls on usual care was included in the model (Supplementary Table 17). Only biomarkers with indicated differences between study participants and controls based on 95% confidence intervals (HbA1c, systolic blood pressure, HDL cholesterol and body weight) were included in the base case scenario.

Mean HbA1c increases over time in patients with type 2 diabetes^60,61^. A linear drift of HbA1c by 0.15% per year (National Glycohemoglobin Standardization Program [NGSP] units) was therefore expected, in agreement with previous reports^60,61^. This drift was assumed to start after three years in study participants, as the HbA1c reduction was maintained, and even enhanced, beyond one year in this group. In control patients on usual care, the drift was assumed to start after one year, in accordance with the observed changes in those individuals.

We also analyzed cost-effectiveness for when the tool is used at least monthly or at least bimonthly, and also assessed the sensitivity to changes in key parameters and assumptions (see Sensitivity analysis).

### Healthcare costs used in the cost-effectiveness analysis

Healthcare costs related to diabetic complications were based on a recent report^60^ and adjusted to 2020 price levels using the monthly consumer price index for healthcare from Statistics Sweden. Costs were converted from SEK to USD using the 2020 average exchange rate (9.3 SEK per USD) at the Riksbank of Sweden.

Analyses of treatment costs were based on information from the study participants on drug usage, including daily dose (Supplementary Table 19). Unit costs of drugs and consumables were in accordance with pharmacy sales price (the Dental and Pharmaceutical Benefits Agency database).

### Expenses related to the tool

The yearly expenses related to the tool consist of server cost ($0.13 per user), email expenses ($0.23 per user), as well as administration and technical personnel for user requests, continuous updates and improvements to the tool ($7.12 per user). These numbers are based on current actual costs for providing the service to 50,000 users (including the participants in the present study and in an international health promotion program conducted by academic institutions). The costs for developing the tool was not included in the analysis, as it is already in place and ready to use.

### Productivity loss

Costs related to productivity loss were based on a recent study on absence from work because of diabetic complications^62^, as well as monthly salary data from Statistics Sweden. The yearly production value was calculated as the average monthly salary, multiplied by 1.4 to account for social expenses, and amounted to $63,514 per person. This was applied in the model for individuals up to 65 years of age.

### Utility

Utility weights related to the health states in the model were in accordance with previously reported data^60^.

### Hypoglycemic event rates

Utility weights related to the health states in the model were in accordance with previously reported data^60^.

The estimated yearly event rates for mild and severe hypoglycemia were 2.6 and 0.02, respectively, per patient using insulin, and 2.0 and 0.005, respectively, per insulin-naïve patient. These data were based on a recent study^30^ and weighted in proportion to the number of patients in the cohort using insulin.

### Statistics

The two primary endpoints were:

The change of HbA1c from first to second visit compared between participants randomized to wait and randomized to access the tool.

The standard interval between first and second visit was 12 weeks. All participants were included in the analysis, independent of medication change or frequency of using the tool, except those lost to follow-up between first and second visit, from whom no data could be obtained. The change of HbA1c was compared between randomization groups using a two-sided independent *t*-test. The sample size, 142 participants in each of the randomization groups, was calculated to ensure at least 80% power at alpha = 0.05 to detect a significant difference between the groups, assuming that the true treatment effect of the tool is 2 mmol/mol over 12 weeks with a standard deviation of 6 mmol/mol for the change of HbA1c. (The standard deviation for the change of HbA1c during 12 weeks was estimated from patients in the ANDIS cohort with baseline HbA1c ≥ 52 mmol/mol.)

The change of HbA1c from baseline to end of follow-up in participants using the tool as recommended compared to matched controls.

Baseline for study participants was defined as HbA1c before getting access to the tool, which was the first visit for those randomized to immediate access and the second visit for those who were initially randomized to wait. If participants were lost to follow-up or changed glucose-lowering medicines during the observation period, then data from the last visit with unchanged medicines was used for analysis (the reported average follow-up time in the study refers to the time from baseline to last visit included in the analyses, i.e. the actual period that was being analyzed). No imputation was done. Recommended usage refers to biweekly usage or more during at least a one-year time frame in the observation period (a patient may e.g. use it biweekly during the first year and then less frequently, but also low-activity periods were included in the analyses to investigate the effects over the entire follow-up). Only matched controls with no registered changes to glucose-lowering medication during the observation period were used. The change of HbA1c was compared between study participants and matched controls (1:2 ratio) by a two-sided independent *t*-test. We needed 24 participants using the tool as recommended and 48 matched controls to have 80% power at alpha = 0.05 to detect a significant difference between the groups, assuming that the true treatment effect of the tool is 5 mmol/mol with a standard deviation of 7 mmol/mol for the change of HbA1c. (The standard deviation for the change of HbA1c per year was estimated from observations of patients in ANDIS with baseline HbA1c ≥ 52 mmol/mol).

Secondary endpoints included the change of secondary variables from baseline to end of follow-up between study participants using the tool as recommended and controls. For controls, data on body weight, blood pressure, total cholesterol, HDL cholesterol, LDL cholesterol, and triglyceride levels during corresponding time from index date were obtained via ANDIS and the Swedish National Diabetes Registry, and data on fasting glucose, HOMA2-IR, HOMA2-B, fat mass and muscle mass were obtained from longitudinal measurements of ANDIS patients at the Clinical Research Center, Malmö, Sweden. Corresponding analyses were conducted for patients with less-frequent usage. The differences between study participants and controls were compared using independent *t*-tests and are presented as averages with 95% confidence intervals.

The association between the number of themes completed and changes of HbA1c, body weight, HOMA2-IR and HOMA2-B, respectively, was analyzed using linear regression.

Change of IPAQ score between initial and final test was analyzed in study participants by paired comparisons and presented as a point estimate with 95% confidence interval.

The distribution of responder and non-responder sum scores for each BCT was analyzed by a chi-square test.

The association between the number of abstract and concrete questions, respectively, and change of HbA1c from baseline was analyzed using linear regression and presented as beta coefficients with 95% confidence intervals.

Mediation analysis was performed using Hayes’ Process macro^63^ v4.0 in SPSS v26. This analysis was undertaken with the hypotheses that change of body weight as well as change of insulin resistance partially mediate the relationship between usage of the tool (measured as number of completed themes) and HbA1c improvement. The analysis gives estimates and confidence intervals of the indirect effect on HbA1c improvement mediated via change of body weight or change of insulin resistance. BMI at baseline and sex were used as moderators in the analysis of body weight, and baseline HOMA2-IR and sex were used as moderators in the analysis of insulin resistance. Data are decomposed into (1) effect of the independent variable on the outcome variable, (2) effect of the independent and mediator variable, respectively, on outcome, and (3) the total effect of the independent variable on the outcome, the indirect effect via the mediator and the fraction of the total effect that is estimated to be an indirect effect.

Summary statistics are generally presented as point estimates with 95% CI. The widths of the intervals have not been adjusted for multiplicity. Statistical analyses were performed using SPSS (v25 and v26).

### Reporting summary

Further information on research design is available in the Nature Research Reporting Summary linked to this article.

## Supplementary information

Supplementary material revised clean

Supplementary Data 1

Reporting Summary

## Data Availability

The data that support the findings of this study are available from the corresponding author upon reasonable request for non-commercial purposes. The trial protocol is appended as Supplementary Data 1.
