# Supplementary material for: Effect of self-managed lifestyle treatment on glycemic control in patients with type 2 diabetes
Source: NPJ Digit Med. 2022 May 11;5:60. doi: 10.1038/s41746-022-00606-9 (PMC9095642; doi:10.1038/s41746-022-00606-9)
Supplement: Supplementary file 1 — Supplementary material revised clean [file 41746_2022_606_MOESM1_ESM.pdf]

Supplementary Appendix

Effect of self-managed lifestyle treatment on glycemic control in patients with type 2 diabetes

TABLE OF CONTENTS

Supplementary discussion: cost-effectiveness analysis 3
Supplementary Figure 1. 6
Supplementary Figure 2. 7
Supplementary Figure 3. 8
Supplementary Table 1. 9
Supplementary Table 2. 10
Supplementary Table 3. 11
Supplementary Table 4. 12
Supplementary Table 5. 13
Supplementary Table 6. 17
Supplementary Table 7. 18
Supplementary Table 8. 19
Supplementary Table 9. 20
Supplementary Table 10. 21
Supplementary Table 11. 22
Supplementary Table 12. 23
Supplementary Table 13. 24
Supplementary Table 14. 25
Supplementary Table 15. 26
Supplementary Table 16. 27
Supplementary Table 17. 28
Supplementary Table 18. 29
Supplementary Table 19. 30
Supplementary Table 20. 31
Supplementary Table 21. 32

SUPPLEMENTARY DISCUSSION: COST-EFFECTIVENESS ANALYSIS

DETAILED RESULTS OF THE BASE CASE SCENARIO

The baseline characteristics and changes of relevant biomarkers were used to estimate the future risk of complications in patients using the tool biweekly, monthly and bimonthly and patients on usual care, respectively (Supplementary Figure 3). These data, together with information on costs for glucose-lowering treatment and diabetic complications, productivity loss and operating expenses was used to model the overall cost-effectiveness of the tool compared with usual care during a simulated 20-year time horizon (Supplementary Table 20).

BASECASE SCENARIO

The incremental gain of QALYs in users of the tool is mainly attributed to increased survival, improved quality-of-life, and reduced BMI. The changes in productivity loss had only a small effect on overall cost increments, which is a result of the mean baseline age being 62 years, i.e. close to the age of retirement.

Overall, the tool was dominant over usual care (more effective and less costly). In the base case scenario, the total estimated cost-savings amounted to $4,116 per person over 20 years compared to usual care.

SENSITIVITY ANALYSIS

To study how sensitive the model results are to changes in key parameters and assumptions, we tested eight different scenarios in addition to the base case scenario. These include:

1,2) A 10-year and a 40-year time horizon

Time horizon is an important parameter, as diabetic complications develop over extended time. While a time horizon of 20 years was used in the base case scenario, we also examined the effects over 10 and 40 years, respectively. In the 40-year scenario, it was assumed that treatment was intensified with additional basal insulin of 0.1 IU per kg of body weight when HbA1c reached a threshold level of 10.1% (87 mmol/mol). This was based on observations of the average HbA1c levels for initiation of insulin therapy in the ANDIS registry. The anticipated effects of insulin treatment on HbA1c and BMI was based on previously reported data.[30] These considerations were not relevant for the other time horizons, as average HbA1c did not drift to the threshold level in this cohort during shorter time periods.

Compared with the base case scenario, the 10-year time horizon resulted in lower QALY gains and cost savings, because fewer long-term complications are taken into account. Increasing the time horizon from 20 to 40 years did not have a major impact on total costs and QALYs, mainly because of discounting effects and limited survival during the entire 40-year period.

3) Inclusion of changes in HbA1c but not secondary variables

As HbA1c was the primary study variable, we investigated a scenario where no changes of secondary variables were included in the model. In this scenario, cost savings were similar to the base case results, while the QALY gain was lower, mainly because changes in quality-of-life and BMI were not considered.

4) Linear increase of HbA1c from first year in both users and controls

Recent meta-analyses have shown that the average metabolic response to digital lifestyle interventions declines after six months.[7] The continuous improvement of HbA1c observed during the entire follow-up period in the present study suggests that the tool leads to more sustainable effects. In the base case, a linear increase of HbA1c (0.15% per year) was assumed to start after three years in users of the tool and after one year in controls, an assumption that was based on the study observations. We analyzed also a more conservative scenario, in which a similar HbA1c increase over time, starting after the first year, was assumed in both groups. This resulted in a change of cost-savings from $4,116 to $3,208 per user over 20 years.

5) **Inclusion of all secondary variables**
The base case scenario included only variables with indicated differences between study participants and controls, based on 95% confidence intervals. This approach is often applied in cost-effectiveness analyses in order to remain conservative. It could, however, be argued that all point estimates should be used in the model. We therefore examined a scenario that also included the observed mean differences of total cholesterol, LDL cholesterol and triglycerides between study participants and controls. This did not have a noticeable impact on the results.

6) **Exclusion of BMI-related effects on quality-of-life.**
The base case scenario included both the direct effects on quality-of-life, as measured by EQ-5D-5L, and indirect effects related to decreased body weight (reduced body weight in patients with type-2 diabetes, as observed in the study, is generally associated with a positive change in quality-of-life).[55] As this could potentially lead to an overestimation of the gain in quality-of-life, we studied a scenario where the model was adjusted to exclude any indirect BMI-related quality-of-life effects and only consider the direct, measured effects. The model results were robust in this respect, and the gain of QALY was only slightly decreased.

7) **Effects based on usage at least once per month including only HbA1c**
We analyzed the cost-effectiveness when the tool was used at least once per month but included only changes in HbA1c, no secondary variables (corresponding to scenario 3 for recommended usage).

8) **Effects based on usage at least once every other month including only HbA1c**
We analyzed the cost-effectiveness when the tool was used at least every other month but included only changes in HbA1c, no secondary variables (corresponding to scenario 3 above).

In each of the scenarios of the sensitivity analysis, the tool resulted in cost savings and more QALYs compared to usual care (Supplementary Table 21). The hypothetical maximum yearly operating expenses of the tool in order to remain cost-saving ("break-even cost") was calculated as total cost savings divided by total remaining life expectancy (ranging from 7.8 to 12.2 years in the different scenarios). The break-even cost varied between $110 and $411 per patient ($369 in the base case scenario), which is well above the actual cost of $7.5. Non-users do not incur any costs and were therefore not included in the models.

The cost-effectiveness analysis was based on Swedish cost data. Given the low operating expenses and assuming that similar metabolic outcomes can be obtained in other contexts, the main conclusions should be applicable to other country-specific settings as well. Since the results indicated that the tool would be cost-saving up to yearly operating expenses of approximately $300 per user, there is room for implementation costs and language adaptations (beyond the current English and Swedish versions) while still obtaining cost-saving outcomes.

CLINICAL CONTEXT

Cost-effectiveness models as the one used here are based on several assumptions that make exact estimations uncertain. In this case, however, a key driver of the results is the low marginal cost of offering the tool to patients. This means that also modest benefits in terms of reducing very costly diabetes complications will make the tool cost-effective. The cost per patient can be put into perspective by comparing it to the annual expenses of metformin, which are at least 10 times higher. This is particularly important considering the large unmet clinical need for scalable solutions to support lifestyle self-management, not least in low-income settings. The intention is to make the tool available to users via 1) healthcare providers (in conjunction with other lifestyle management activities) and 2) direct access by patients.

From the onset, the aim has been to develop a tool that is freely available to the individual patient. Many digital solutions that are technically scalable still fail to reach large patient groups because of associated costs. Some solutions

require expensive coaching or enhanced healthcare support that limit broad applicability. Several tools also apply user fees or require patients to give back health data to be used for third party purposes.

For many drug and lifestyle interventions it is critical to compare the costs and/or side effects versus overall efficacy. In this case, the tool is provided at low cost per user and has no known negative side effects. It means that even if the tool is not used by all patients, it could still be of considerable clinical importance. A clinician or healthcare provider could choose to 1) provide the tool only to patients with MOD characteristics (or apply a BMI cut-off), which is likely to give substantial benefits in that population, analogous to a tailored drug, or 2) offer it to all patients with type 2 diabetes, as any additional patient incurs a minimal cost and those who use it regularly are likely to get overall improvements.

SUPPLEMENTARY FIGURES

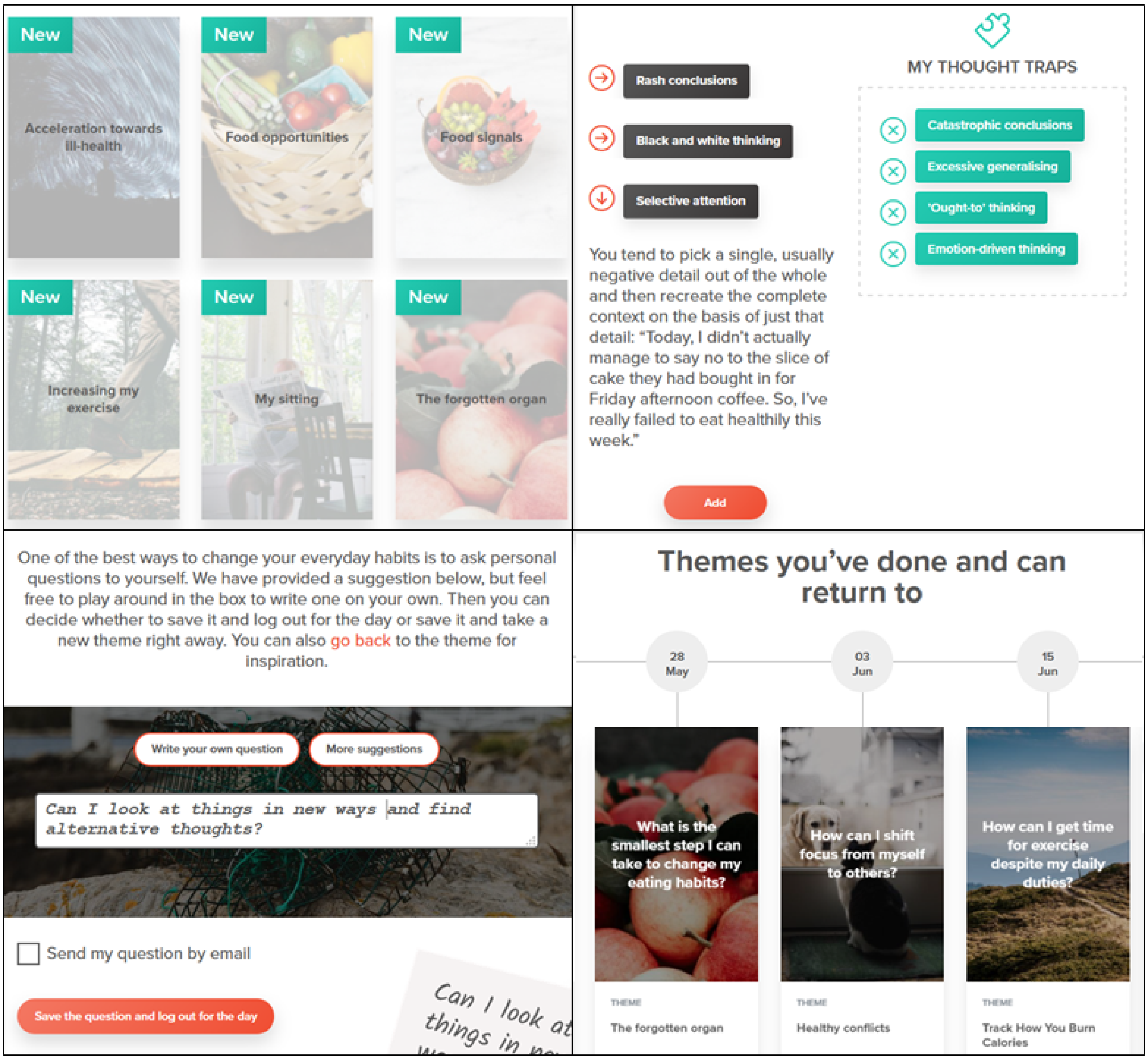

Supplementary Figure 1. User journey on the tool.

Upper left: At each login, participants can choose one of 80 different themes. Themes are presented with a brief introductory text that is displayed when clicking on the theme title.
Upper right: A theme consists of tests in order to explore current habits, texts with health information and exercises to learn behavior change techniques.
Lower left: At the end of each theme, participants ask a question to themselves on how to implement the content and insights from the theme. They are encouraged to reflect on their question in daily life, explore different options and return to the tool within two weeks.
Lower right: When returning, participants choose a new theme. They can also follow their personal journey and the questions they asked, and revisit themes they have previously done and found valuable.

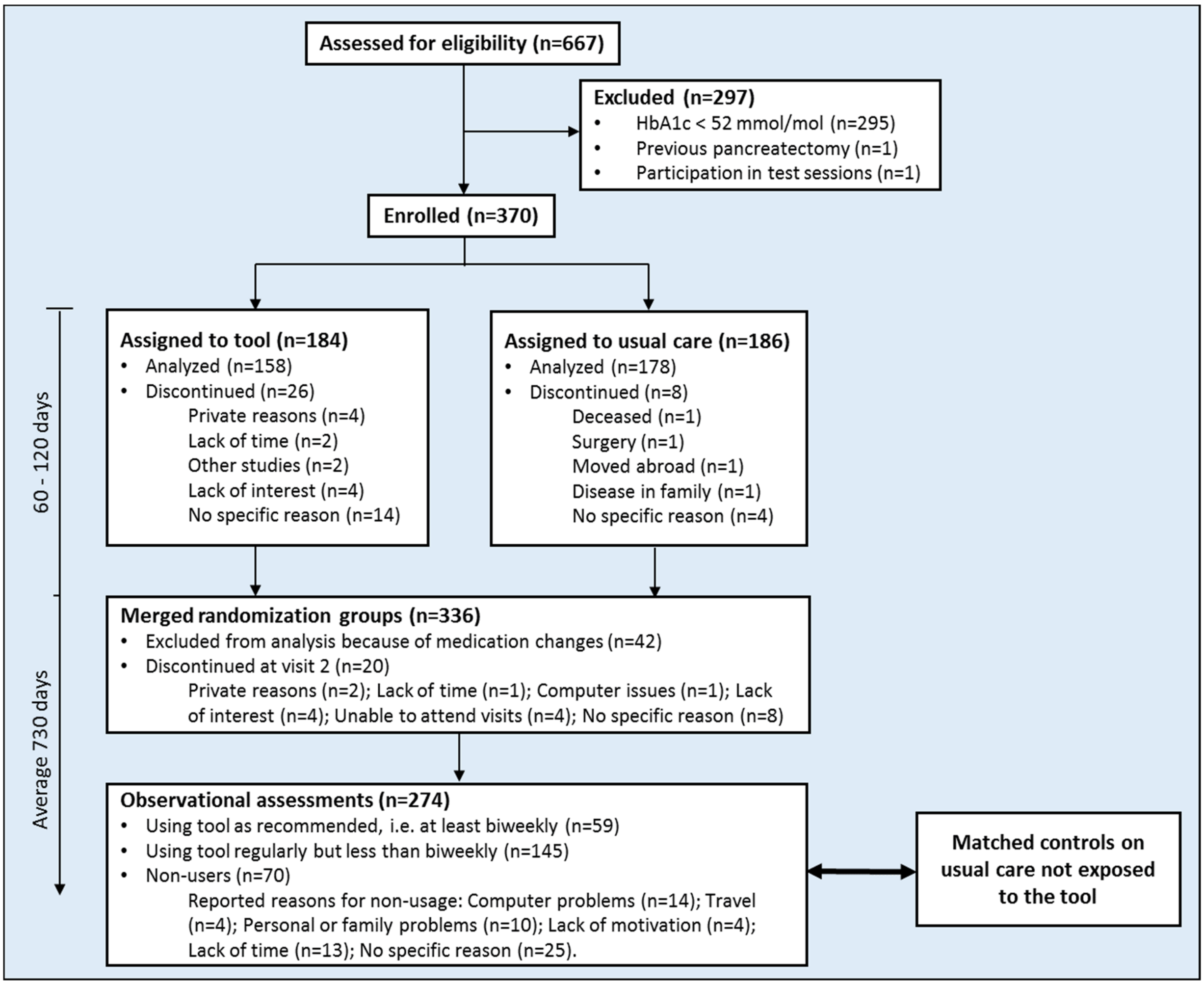

Supplementary Figure 2. Study profile as CONSORT diagram.

A total of 370 individuals were randomized to usual care or to access the tool. Twenty-six of the participants randomized to the tool and eight on usual care discontinued between first and second visit (reported reasons in the figure). Change of HbA1c from first to second visit was compared between randomization groups.

After the second visit, the randomization groups were merged to enable all participants to use the tool during an extended open-label period of 730 days on average. The change of HbA1c from baseline was compared between participants and matched controls on usual care. Baseline for study participants during the long-term assessment period was defined as HbA1c before accessing the tool, which was the first visit for those randomized to immediate access and the second visit for those initially randomized to wait. Twenty participants who had been on wait discontinued at the second visit and did therefore not provide any data beyond baseline, and 42 participants were excluded from analysis because of changed glucose-lowering medication just after baseline assessments. If participants changed glucose-lowering medicines during the follow-up period, then data from the last visit with unchanged medication was used for analysis.

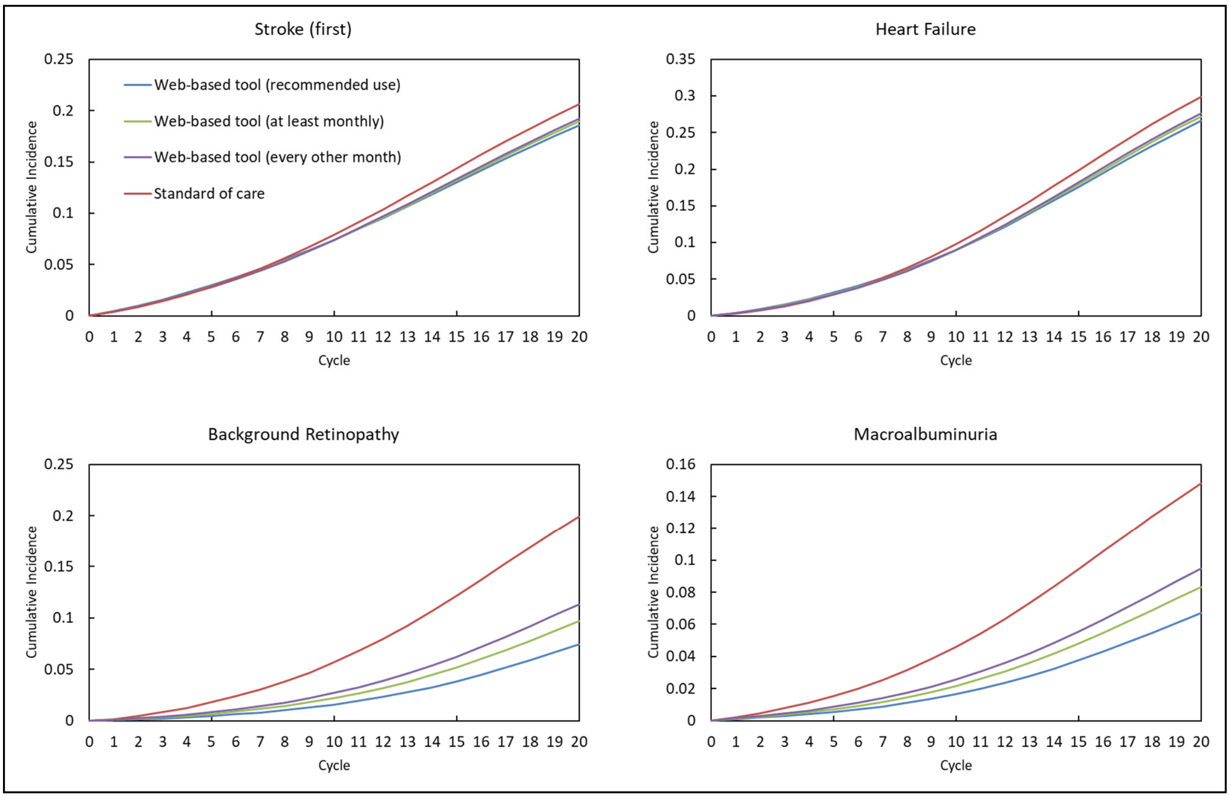

Supplementary Figure 3. Estimated incidence of microvascular and macrovascular complications.

Baseline characteristics and changes of relevant biomarkers in participants using the tool and matched controls were used to model the future risk of complications. Estimated cumulative incidence data for first stroke, heart failure, background retinopathy and macroalbuminuria are shown for usage of the tool biweekly, at least monthly and at least every other month, respectively. Each cycle in the model corresponds to one year.

SUPPLEMENTARY TABLES

Supplementary Table 1. Demographic and baseline characteristics of patients from ANDIS in longitudinal analysis of psychological factors and glycemic control. *

Characteristic	Cohort (n=195)
Male sex – no.	118 (60.5%)
Age – years	64.7 (7.8)
Diabetes duration – years	4.1 (3.4)
Body mass index**	29.8 (4.7)
Glycated hemoglobin level – mmol/mol***	46.7 (7.5)
Glucose-lowering medication – no.	
None	52 (26.7%)
Oral only	129 (66.1%)
Oral and insulin	13 (6.7%)
Insulin only	1 (0.5%)
Socioeconomic status – no.	
Employed	57 (31.7%)
Unemployed	5 (2.8%)
Retired	91 (50.6%)
Sick-leave > 3 months	12 (6.7%)
Taking care of own household	2 (1.1%)
Highest education – no. ****	
Basic level	44 (24.4%)
Medium level	60 (33.3%)
College/University	47 (26.1%)

* Data are n (%) or mean (SD). Data on socioeconomic status and education were not available from all patients.
**The body mass index is the weight in kilograms divided by the square of the height in meters.
***Glycated hemoglobin level (HbA1c) was analyzed according to International Federation of Clinical Chemistry (IFCC) standard.
****Basic level refers to up to 9 years of education; medium level is up 12 years of education.

Supplementary Table 2. Association between questionnaire scores and HbA1c across visits. *	
**Scale**	**Beta coefficient from linear model (n=188)**
Perceived competence for diabetes	-0.28 (95% CI -0.47 to -0.09) **
Appraisal of diabetes	0.63 (95% CI 0.25 to 1.0)
Autonomous (intrinsic) motivation	-0.08 (95% CI -0.27 to 0.10)
Controlled (extrinsic) motivation	-0.06 (95% CI -0.24 to 0.12)
Influence of life view on health-related habits	-0.56 (95% CI -1.1 to -0.05)

*The association between questionnaire scores and HbA1c across time (average 32 months) was analyzed using a linear model in which values from each visit were included as discrete observations and grouped by study subject. The unstandardized beta coefficient from the linear model is reported with 95% confidence intervals (CI).
**Negative beta coefficient implies that increased questionnaire score is associated with reduced HbA1c over time.

Supplementary Table 3. Demographic and baseline characteristics of patients taking parts in interviews. *	
**Characteristic**	**Interview cohort (n=22)**
Male sex – no.	12 (54.5%)
Age – years	65.8 (8.3)
Diabetes duration – years	2.9 (3.0)
Body mass index**	29.6 (6.5)
Glycated hemoglobin level – mmol/mol	46.2 (7.0)
Glucose-lowering medication – no.	
None	8 (36.4%)
Oral only	13 (59.1%)
Oral and insulin	1 (4.5%)
Insulin only	0 (0.0%)
Socioeconomic status – no.	
Employed	6 (27.3%)
Unemployed	1 (4.5%)
Retired	11 (50.0%)
Sick-leave > 3 months	3 (13.6%)
Taking care of own household	1 (4.5%)
Highest education – no. ***	
Basic level	8 (36.4%)
Medium level	4 (18.2%)
College/University	10 (45.4%)

* Data are n (%) or mean (SD).
**The body mass index is the weight in kilograms divided by the square of the height in meters.
***Basic level refers to up to nine years of education; medium level is up 12 years of education.

Supplementary Table 4. Description of behavior change techniques (BCT). *	
**BCT no.****	**Description**
1	Provide information on consequences of behavior in general
2	Goal setting (focusing on behavior rather than outcome)
3	Action planning (linking behavioral responses to situational cues)
4	Barrier identification/problem solving (reflecting on barriers and how to overcome them)
5	Prompt generalization of target behavior (make behaviors an integrated part of life)
6	Prompt self-monitoring of behavior (in the form of a diary on the tool)
7	Prompt focus on past success
8	Provide feedback on performance (on the tool by providing data and summaries on own recorded behavior)
9	Provide information on where and when to perform the behavior
10	Provide instruction on how to perform the behavior (on the tool in the form of concrete methods and advice on physical activity, stress management etc.)
11	Model or demonstrate the behavior (on the tool in the form of patient examples reflecting a range of life situations and problem areas)
12	Teach to use prompts / cues (identifying environmental signals to remind individuals to perform a behavior)
13	Environmental restructuring (e.g. removing unhealthy food, facilitating physical activity in daily life)
14	Use follow-up prompts
15	Plan social support /social change
16	Prompt anticipated regret (expectations of future feelings in cases of performance or non-performance of the behavior)
17	Prompt self-talk
18	Prompt use of imagery (visualizing successful performance of behavior)
19	Relapse prevention / coping planning (planning how to maintain a changed behavior)
20	Stress management
21	Emotional control training
22	Time management
23	General communication skills training (e.g. listening skills to support relationships and coping with conflicts)
24	Stimulate anticipation of future rewards

*The behavior change techniques (BCT) included in themes on the tool are based on the taxonomy proposed by Michie and colleagues[50] and adapted to digital form.
**A total of 24 BCT were used altogether out of the 40 techniques[50] in the taxonomy.

Supplementary Table 5. Description of themes on the tool.					
**Theme title**	**BCT1***	**BCT2**	**BCT3**	**BCT4**	**Theme description**
A simple change	9	12	6	2	Exercise on healthy choices combined with texts on Mediterranean food.
A simple change - Go deeper	1	17	5	3	Texts on saturated/unsaturated fat and how to change the way of thinking about abstaining and indulging oneself.
Acceleration towards ill-health	11	20	22	5	Exercise on coping with the pace of life combined with an exercise on mindful eating.
Are you fooling yourself?	5	6	11	17	Exercise on common self-deceptions related to health and diabetes.
Being in the moment	8	20	11	18	A test on attentiveness in daily life combined with an exercise on mindfulness.
Being in the moment – Go deeper	20	11	10	18	Exercises on mindfulness.
Chasing time thieves	12	8	2	22	Exercise on prioritizing among activities and practical tips to get more time for health.
Chasing time thieves– Go deeper	17	21	15	11	Texts on life balance and on making room for healthy habits.
Daily presence	8	18	17		A test on mindfulness in daily life combined with texts on how to be more attentive.
Daily presence - Go deeper	24	17	5	18	Texts on self-awareness.
Daily thankfulness	17	15	7	3	Exercise on thankfulness and attitude to life.
Daily thankfulness– Go deeper	7	3	2	4	Texts on coping with disease.
Embellish breakfast	8	2	3	1	Exercise on breakfast habits.
Embellish breakfast - Go deeper	1	2	3	17	Texts on beverages, sugar and sweeteners, and practical tips on nutrition.
Exercise within reach	6	3	9	12	Exercise on how to increase daily exercise combined with texts on intentions and habits.
Exercise within reach - Go deeper	1	17	2		Text on the causes of diabetes.
Food opportunities	1	8	19	17	Exercise on eating habits combined with texts on practical tips on nutrition and various ways to prepare food.
Food opportunities - Go deeper	1	24	17	4	Texts on intermittent fasting, why different diets are debated so intensely, and a historic review of the discovery of insulin.
Food problems	7	5	6	17	Exercise on coping strategies for eating problems.
Food problems - Go deeper	1	24	5	17	Texts on glycaemic index, bread habits and why health information can be so hard to digest.
Food signals	12	9	13	3	Exercise on identifying and removing cues that trigger unhealthy eating, combined with a structured approach for changing eating behaviors.
Food signals - Go deeper	14	9	12	3	Texts on how to eat "as simple as possible, but not simpler", using vegetables, berries, fish etc., without extra sugar, salt and fat.
Free choice or fate	17	11			Exercise to stimulate reflection on opportunities and limitations to affect personal health and to cope with disease.
Green ideas	1	17			Exercise on exploring new ways to eat more vegetables combined with texts on practical tips on nutrition and why vegetables are healthier than vitamin supplements.
Green ideas – Go deeper	8	4	2	3	Texts on what happens in the body when we eat and why it matters to reflect on lifestyle.

Health through acceptance	21	17	11	3	Exercise on how to practice acceptance in real life to better cope with distress and factors beyond control.
Healthy conflicts	21	23	17	3	Exercise on managing conflicts and how conflicts could affect health.
Healthy food	3	10	2	7	Exercise on improving eating habits and practical tips on healthy food.
Healthy food – Go deeper	1	11	17		Text on how to prevent or treat type 2 diabetes.
How I eat	8	1	12	5	Test on eating habits along three dimensions, combined with texts on fast food vs. slow cooking, the attitude to eating and on changing eating behavior.
How I eat - Go deeper	5	12	1	3	An exercise on mindful eating combined with a text about what has been shown in studies on different dietary patterns and overall eating behavior.
Increasing my exercise	2	1	8	5	Exercise on increasing physical activity, combined with texts on how to better cope with mental problems via physical activity and different forms of motivation to exercise.
Increasing my exercise - Go deeper	1	19	2	3	Texts on the influence of genetics on the effect of physical activity and step-wise methods to achieve health-related goals.
Me and my health	15	10	17	18	Exercise on how the balance between myself and others can affect health.
Me and others	21	15	8	17	Exercise on different forms of motivations.
Me and others – Go deeper	17	7			Exercise on the influence of relationships and loneliness on health.
My core questions – Art**	17	18	13	11	Exercises and texts on the different aspects of existential health proposed by the WHO
My core questions – Buber**	18	17			Exercises and texts on the different aspects of existential health proposed by the WHO
My core questions – Wholeness**	17	8	18	20	Exercises and texts on the different aspects of existential health proposed by the WHO
My core questions – Harmony**	17	18	21	24	Exercises and texts on the different aspects of existential health proposed by the WHO
My core questions –Harmony** – Go deeper	18	21	17	15	Exercises and texts on the different aspects of existential health proposed by the WHO
My core questions – Mandela**	15	17	24		Exercises and texts on the different aspects of existential health proposed by the WHO
My core questions - Dag Hammarskjöld's Markings**	17	18	21		Exercises and texts on the different aspects of existential health proposed by the WHO
My core questions – Dag Hammarskjöld's Markings - Go deeper**	17	18	11		Exercises and texts on the different aspects of existential health proposed by the WHO
My core questions – Frankl**	11	17	24		Exercises and texts on the different aspects of existential health proposed by the WHO
My core questions – Frankl – Go deeper**	11	17	24		Exercises and texts on the different aspects of existential health proposed by the WHO
My core questions – Meditation**	11	10	21		Exercises and texts on the different aspects of existential health proposed by the WHO
My core questions – Metaphors**	18	17			Exercises and texts on the different aspects of existential health proposed by the WHO
My core questions – Strength**	17	18			Exercises and texts on the different aspects of existential health proposed by the WHO

My core questions – Purpose**	23	15	10	17	Exercises and texts on the different aspects of existential health proposed by the WHO
My core questions – Rest**	18	17			Exercises and texts on the different aspects of existential health proposed by the WHO
My core questions – Silence**	21	18	13	17	Exercises and texts on the different aspects of existential health proposed by the WHO
My core questions – Closeness**	21	18	17		Exercises and texts on the different aspects of existential health proposed by the WHO
My core questions – Socrates**	17	18			Exercises and texts on the different aspects of existential health proposed by the WHO
My core questions – Socrates – Go deeper**	17	21	18		Exercises and texts on the different aspects of existential health proposed by the WHO
My core questions – Trust**	18	21			Exercises and texts on the different aspects of existential health proposed by the WHO
My core questions – New Steps**	17				Exercises and texts on the different aspects of existential health proposed by the WHO
My sitting	8	3	1		Test on current sedentary behavior combined with a text on how to stand up for better health.
My sitting – Go deeper	1	3	12	17	Texts on different types of diabetes, diabetes complications and why good intentions have such a bad reputation.
Relationships and health	11	10	21	17	Exercise to stimulate reflection on the influence of social context on health.
Relationships and health – Go deeper	14	1	15	17	Text on loneliness and health.
Reducing noise	12	17	22	11	Exercise on how to reduce distractions, combined with strategies for overcoming distractions and coping with stress.
Reducing noise – Go deeper	13	17	10	5	Text to stimulate reflection on what is essential in life and how current priorities affect health.
Some Nobel Prize Winners	1	10	5		Exercise on decision-making inspired by Daniel Kahneman's research, including influence of biases and small daily decisions on health and disease coping.
Strengths	8	7	6		Test on how to use strengths from other areas to cope with disease and lifestyle.
Strengths – Go deeper	15	7	16	3	Additional tests on how to use strengths from other areas to cope with disease and lifestyle.
Thinking about how you think	21	18	19	1	Exercise on how thoughts may affect lifestyle, distress and disease coping.
The forgotten organ	1	17			Texts on nutrition, gut flora and health, what is known and what is currently uncertain.
Thought traps	21	3	11	6	Techniques to examine and challenge common thought traps, e.g. selective attention and biases, that may affect health.
Time and health	22	17	6	10	Exercise on how attitudes to time, including the extent of focus on the past, present and future, affect health and quality of life.
Time prioritization	22	8	20	18	Test on time management, combined with texts on how to prioritize health in daily life and on the art of saying no.
Time prioritization – Go deeper	24	2	3	17	Text on why healthy habits can be so difficult to prioritize.
To be present	20	11	10	18	Mindfulness exercises.
To be present – Go deeper	20	11	10	18	Mindfulness exercises.

Track How You Burn Calories	8	1	17		Test on the weekly amount of physical activity, combined with texts on how physical activity can improve stress coping.
Track How You Burn Calories – Go deeper	1	12	2	3	Texts on methodologies to establish new habits and on the pathophysiology and complications of type-2 diabetes.
Trust	16	24	4	2	Exercise on making life-changing decisions combined with concrete methods to support sustainable lifestyle changes.
Trust – Go deeper	8	6	18	15	Texts on loneliness, relationships and health.
Using my time wisely	8	1	10	18	Test on time management combined with texts on what research has shown about the influence of circadian rhythm on glucose control and health.
Using my time wisely – Go deeper	10	20	22	17	Texts on methods to reduce procrastination and how to give more time for healthy habits.

*Up to four behavior change techniques (BCT) were incorporated into each theme. They are ranked according to predominance within the theme.
**Core questions refer to a set of themes that focus on the different aspects of existential health that the World Health Organization (WHO) has proposed.[53,54] These themes aim to stimulate questions on overall life context and how it relates to current habits and disease coping. The themes are more essayistic in style compared with other themes on the tool and may include references to e.g. Socrates, Victor Frankl, Martin Buber, Dag Hammarskjöld and others as starting points to inspire further reflections by the individual user.

**Supplementary Table 6. Demographic and baseline characteristics of participants analyzed in randomization groups. ***		
**Characteristic**	**Tool (n=158)**	**Usual care (n=178)**
Male sex – no.	97 (61.4%)	109 (61.2%)
Age – years	63.7 (9.5)	63 (9.8)
Diabetes duration – years	4.2 (1.4)	4.1 (1.4)
Body mass index**	30.9 (5.2)	31.2 (5.1)
Glycated hemoglobin level – mmol/mol	63.6 (10.7)	62.9 (9.7)
Glucose-lowering medication – no.		
None	7 (4.5%)	7 (4%)
Oral only	103 (66.9%)	116 (66.3%)
Oral and insulin	33 (21.4%)	38 (21.7%)
Insulin only	11 (7.1%)	14 (8%)
Socioeconomic status – no.		
Employed	63 (43.8%)	76 (45.5%)
Unemployed	5 (3.5%)	2 (1.2%)
Retired	71 (49.3%)	78 (46.7%)
Sick-leave > 3 months	5 (3.5%)	8 (4.8%)
Taking care of own household	0 (0%)	3 (1.8%)
Highest education – no. ***		
Basic level	25 (18.2%)	25 (15.4%)
Medium level	44 (32.1%)	50 (30.9%)
College/University	68 (49.7%)	87 (53.7%)

*Data are n (%) or mean (SD). Data on glucose-lowering medication, socioeconomic status and education were not available from all.
**The body mass index is the weight in kilograms divided by the square of the height in meters.
***Basic level refers to up to 9 years of education; medium level is up 12 years of education.

Supplementary Table 7. Key pathophysiological characteristics of Mild Obesity-related Diabetes (MOD).*		
**Characteristic**	**Participants with MOD (30%)**	**Participants without MOD (70%)**
Age – years	57.2 (9.3)	64.3 (8.1)
Diabetes duration – years	3.7 (1.7)	3.8 (1.6)
Body mass index**	35.1 (4.3)	30.1 (4.5)
Glycated hemoglobin level – mmol/mol	63.5 (12.2)	60.5 (8.3)
HOMA2-IR	3.9 (1.7)	3.4 (1.6)
HOMA2-B	62.3 (31.3)	65.5 (30.6)

* Study participants were categorized as MOD and non-MOD, respectively, using the ANDIS clustering methodology.[14] Data are mean (SD).
**The body mass index is the weight in kilograms divided by the square of the height in meters.

**Supplementary Table 8. Demographic and baseline characteristics of participants who discontinued before long-term assessments. ***		
**Characteristic**	**Discontinued (n=54) ****	**Continued (n=316)**
Male sex – no.	36 (67.9%)	194 (61.0%)
Age – years	66.8 (9.8)	62.7 (9.5)
Diabetes duration – years	4.3 (1.5)	4.2 (1.4)
Body mass index***	31.1 (5.2)	31.0 (5.2)
Glycated hemoglobin level – mmol/mol	63.9 (10.7)	63.1 (10.2)
Glucose-lowering medication – no.		
None	0 (0%)	14 (4.5%)
Oral only	32 (65.3%)	206 (66.2%)
Oral and insulin	10 (20.4%)	69 (22.2%)
Insulin only	7 (14.3%)	22 (7.1%)
Socioeconomic status – no.		
Employed	9 (33.3%)	137 (45.8%)
Unemployed	0 (0%)	7 (2.3%)
Retired	18 (66.7%)	138 (46.2%)
Sick-leave > 3 months	0 (0%)	14 (4.7%)
Taking care of own household	0 (0%)	3 (1.0%)
Highest education – no. ****		
Basic level	6 (22.2%)	45 (15.7%)
Medium level	6 (22.2%)	93 (32.4%)
College/University	15 (55.5%)	149 (51,9%)

* Data are n (%) or mean (SD). Data on glucose-lowering medication, socioeconomic status and education were not available from all participants.
**A total of 34 participants (26 randomized to access and 8 randomized to wait) discontinued between first and second visit during the randomization period. Another 20 participants who had been on wait discontinued at second visit and did not provide any observational data beyond baseline. Participants who changed glucose-lowering medication immediately after baseline (n=42) were not included in the follow-up analyses but remained in the study and are therefore not reported as discontinued.
***The body mass index is the weight in kilograms divided by the square of the height in meters.
****Basic level refers to up to 9 years of education; medium level is up 12 years of education.

**Supplementary Table 9. Demographic and baseline characteristics of participants in analyses from baseline to end of follow-up. ***			
**Characteristic**	**Biweekly use or more (n=59)**	**Regular use less than biweekly (n=145)**	**Non-users (n=70)**
Male sex – no.	27 (45.8%)	89 (61.0%)	54 (79.4%)
Age – years	62.1 (8.6)	63.0 (10.4)	61.6 (9.8)
Diabetes duration – years	4.6 (1.8)	5.1 (2.1)	4.1 (1.3)
Body mass index	32.2 (6.5)	30.9 (4.9)	31.5 (5.0)
Glycated hemoglobin level – mmol/mol	62.1 (8.6)	63.0 (10.4)	62.5 (9.4)
Glucose-lowering medication – no.			
None	2 (3.4%)	8 (5.5%)	4 (5.9%)
Oral only	37 (62.7%)	97 (66.4%)	42 (61.8%)
Oral and insulin	14 (23.7%)	30 (20.5%)	16 (23.5%)
Insulin only	4 (6.8%)	8 (5.5%)	6 (8.8%)
Socioeconomic status – no.			
Employed	18 (30.5%)	66 (45.2%)	32 (51.6%)
Unemployed	2 (3.4%)	2 (1.4%)	3 (4.8%)
Retired	30 (50.8%)	65 (44.5%)	24 (38.7%)
Sick-leave > 3 months	4 (6.8%)	5 (3.4%)	3 (4.8%)
Taking care of household	0 (0%)	2 (1.4%)	0 (0%)
Highest education – no.			
Basic level	12 (24.0%)	22 (15.1%)	11 (18.6%)
Medium level	18 (36.0%)	38 (26.0%)	20 (33.9%)
College/University	25 (40.0%)	72 (49.3%)	28 (47.6%)
Baseline physical activity – MET-minutes**	2076 (2114)	2100 (3152)	2851 (2947)

* The pattern of usage was observed in study participants from baseline to end of follow-up. Data are n (%) or mean (SD). Data on glucose-lowering medication, socioeconomic status and education were not available from all participants. Biweekly usage refers to biweekly usage during at least a one-year time frame in the follow-up period (a patient may e.g. use it less than biweekly during the first year and then use it biweekly during the second year, but also low-activity periods were included in the analyses to investigate the effects over the entire follow-up).
** Physical activity measured at baseline by IPAQ

Supplementary Table 10. Weighted descriptive statistics when adjusting for potential confounders related to frequent usage of the tool*				
**Characteristic**	**Non-users (n =90.5)**	**Biweekly users (n =86.3)**	**SMD** weighted**	**SMD** unweighted**
Male sex – no.	67 (74.6%)	52 (60.8%)	0.27	0.95
Age – years	61.4 (9.7)	60.2 (9.9)	0.12	0.10
Diabetes duration – years	4 (1.4)	4.2 (1.4)	0.13	0.19
Body mass index	31.9 (5.2)	32.5 (6.1)	0.12	0.46
Glycated hemoglobin level – mmol/mol	62.6 (9.5)	62.0 (7.9)	0.07	0.07
Glucose-lowering medication no. with insulin treatment ***	28 (31.2%)	26 (30.7%)	0.01	0.12
Socioeconomic status – no.			0.24	0.42
Employed	43 (51.3%)	30 (41.5%)		
Unemployed	9 (10.5%)	12 (17.1%)		
Retired	32 (38.2%)	30 (41.5%)		
Highest education – no. with college/university education ****	39 (48.5%)	28 (38.5%)	0.20	0.15

*Analysis weights based on propensity scores were used to adjust for potential confounders related to frequent usage of the tool. The weights are based on the probability of using the tool as recommended and are applied to statistically adjust the composition of both the group of non-users and the group using the tool biweekly to estimate the mean difference between groups if they were more comparable in terms of baseline characteristics. (The number of individuals in adjusted groups will not always be integers because of the applied weights). Data are n (%) or mean (SD).

**The weights were optimized to minimize the standard mean difference (SMD) between the groups (a smaller SMD indicates more comparable groups). The unweighted SMD and the SMD when applying weights are given for each baseline variable.

***The number of categories was reduced to enable weight optimization (one category refers to no treatment or oral only; the other refers to insulin with or without combined oral treatment).

****The number of categories was reduced to enable weight (one category refers to basic or medium level; the other refers to college/university education).

Supplementary Table 11. Descriptive statistics of study participants and matched controls. *			
**Characteristic**	**Study participants**	**Matched controls on usual care****	**SMD*****
Participants using the tool as recommended and matched controls (1:2 ratio)			
Age – years	60.7 (8.5)	60.5 (8.1)	0.025
Body mass index****	34.3 (7.0)	33.9 (6.7)	0.058
Glycated hemoglobin level – mmol/mol	61.9 (8.3)	61.1 (8.9)	0.095
Participants using the tool regularly but less than recommended and matched controls (1:2 ratio)			
Age – years	63.9 (9.3)	63.2 (8.8)	0.076
Body mass index	30.4 (4.8)	30.4 (4.9)	<0.001
Glycated hemoglobin level – mmol/mol	61.9 (9.3)	61.1 (9.7)	0.084

*Matching characteristics for study participants and control patients from ANDIS. Data are n (%) or mean (SD).
**Controls were matched exactly on gender and on Mahalanobis distance based on age, body mass index and glycated hemoglobin level.
***Balance after matching was evaluated using the standardized mean difference (SMD).
****The body mass index is the weight in kilograms divided by the square of the height in meters.

**Supplementary Table 12. Baseline characteristics of study participants using the tool and controls. ***		
**Characteristic**	**Study participants (n=204)**	**Controls**
Diabetes duration – years	5.0 (2.0)	4.5 (1.8)
Fasting plasma glucose (mmol/l)	9.8 (2.2)	8.3 (0.8)
HOMA2-IR	3.1 (1.7)	2.9 (1.1)
HOMA2-B	58.9 (28.2)	101.6 (37.2)
Fat mass (%)	35.3 (8.4)	34.1 (8.8)
Muscle mass (%)	41.4 (5.4)	43.4 (7.7)
Total cholesterol (mmol/l)	4.4 (1.1)	4.8 (1.2)
LDL cholesterol (mmol/l)	2.7 (1.0)	2.7 (1.1)
HDL cholesterol (mmol/l)	1.3 (0.3)	1.2 (0.3)
Triglycerides (mmol/l)	2.0 (1.1)	1.9 (0.9)
Systolic blood pressure (mm Hg)	144 (18)	138 (17)
EQ-5D-5L**	0.84 (0.14)	-

*Table presents baseline data that were not used as matching variables (see Supplementary Table 11) between study participants and controls. Data are means (SD).
**EQ-5D-5L data were only available for study participants. Score ranges from 0 to 1 with higher score indicating better quality-of-life.

Supplementary Table 13. Trajectories of HbA1c progression. *				
**Pattern**	**Biweekly or more (n=59)**	**Monthly or more (n=145)**	**Bimonthly or more (n=204)**	**ANDIS (n=1358)**
Sustained decrease relative to baseline**	26 (50%) ***	42 (39%) ***	57 (39%) ***	213 (16%)
Sustained increase relative to baseline**	1 (2%) ***	7 (7%) ***	12 (8%)	188 (14%)
Oscillatory, predominantly decreasing**	15 (29%)	30 (28%)	36 (24%)	228 (17%)
Oscillatory, predominantly increasing**	10 (19%)	28 (26%)	42 (29%)	273 (20%)
Did not meet analysis criteria	7	37	58	456

*Trajectories of HbA1c progression were analyzed in study participants when the tool was used as recommended (biweekly), at least monthly and at least bimonthly and were also analyzed in patients with type-2 diabetes in ANDIS. Individuals who had a baseline HbA1c at 52 mmol/mol or above and at least three HbA1c measurements over a three-year time frame with no known medication changes were included. Average follow-up period was 632 days in ANDIS and 730 for users of the tool. Data are presented as number of patients (% of total number of patients eligible for analysis).
**Four common patterns of HbA1c trajectories were identified. The number of patients corresponding to each pattern was compared between ANDIS patients and users of the tool using Fisher's exact test.
***Fisher's exact test statistic <0.001.
****Fisher's exact test statistic <0.05.

Supplementary Table 14. Mediation analyses. *	
**Variable**	**Coefficient****
**Analysis of change of body weight (n=258)*****	
**Outcome variable:** Change of body weight	
**Independent variable:** Usage of tool	-0.0398 (95% CI -0.0711 to -0.0085)
**Outcome variable:** Change of HbA1c	
**Independent variable:** Usage of tool	-0.1113 (95% CI -0.2187 to -0.0040)
**Mediator:** Change of body weight	1.152 (95% CI 0.7335 to 1.571)
**Outcome variable:** Change of HbA1c	
**Total effect** (usage of tool on change of HbA1c)	-0.1572 (95% CI -0.2690 to -0.0454)
**Indirect effect** (mediated via change of body weight)	-0.0459 (95% CI -0.0942 to -0.0068)
**% indirect of total effect**	29.2
**Analysis of change of insulin resistance (HOMA2-IR; n=244)******	
**Outcome variable:** Change of HOMA2-IR	
**Independent variable:** Usage of tool	-0.0134 (95% CI -0.0235 to -0.0033)
**Outcome variable:** Change of HbA1c	
**Independent variable:** Usage of tool	-0.1193 (95% CI -0.2233 to -0.0154)
**Mediator:** Change of HOMA2-IR	2.039 (95% CI 0.7491 to 3.329)
**Outcome variable:** Change of HbA1c	
**Total effect** (usage of tool on change of HbA1c)	-0.1467 (95% CI -0.2510 to -0.0423)
**Indirect effect** (mediated via change of HOMA2-IR)	-0.0273 (95% CI -0.0630 to -0.0029)
**% indirect of total effect**	18.6

*Table presents results from mediation analyses with usage of tool, defined as number of completed themes, as the independent variable, change of HbA1c as the outcome variable and change of body weight or change of HOMA2-IR as mediator. Analyses are decomposed into 1) effect of the independent variable on the outcome variable, 2) effect of the independent and mediator variable, respectively, on outcome, and 3) the total effect of the independent variable on the outcome, the indirect effect via the mediator and the fraction of the total effect that is estimated to be an indirect effect. The total effect is not entirely similar between the analyses since the number of participants with available data on body weight and HOMA2-IR at time points corresponding to those of HbA1c measures differed (n=258 and n=244, respectively).

**Point estimates and 95% confidence intervals of regression coefficients.

***Baseline BMI and sex were used as moderators in the model.

****Baseline HOMA2-IR and sex were used as moderators in the model.

**Supplementary Table 15. Analysis of behavior change techniques.**			
**Behavior change techniques (BCT)**	**Responder score***	**Non-responder score**	**Chi-square statistic** with Yates' correction**
1. Provide information on consequences of behavior in general	**1866**	**1462**	**5.6**
2. Goal setting (behavior)	587	575	6.3
3. Action planning	589	486	0.2
4. Barrier identification/problem solving	**294**	**178**	**12.5**
5. Prompt generalization of target behavior	399	363	1
6. Prompt self-monitoring of behavior	394	320	0.3
7. Prompt focus on past success	256	208	0.2
8. Provide feedback on performance	**2277**	**1785**	**7**
9. Provide information on where and when to perform the behavior	357	352	4.1
10. Provide instruction on how to perform the behavior	406	386	2.7
11. Model or demonstrate the behavior	515	756	99.2
12. Teach to use prompts/cues	509	572	22.4
13. Environmental restructuring	198	180	0.5
14. Use follow-up prompts	**68**	**20**	**18.1**
15. Plan social support / social change	**437**	**305**	**6.8**
16. Prompts anticipated regret	**234**	**142**	**9.8**
17. Prompt self-talk	**2314**	**1836**	**5.1**
18. Prompt use of imagery	1016	839	0.3
19. Relapse prevention / coping planning	**244**	**150**	**9.5**
20. Stress management	425	391	1.4
21. Emotional control training	497	378	2.5
22. Time management	288	314	9.7
23. General communication skills training	103	103	1.3
24. Stimulate anticipation of future rewards	578	475	0.3

*Overall scores of responders and non-responders for every BCT across all themes during one year (see also Methods and Supplementary Table 4-5).

**The distribution of responder and non-responder scores was analyzed by chi-square tests to assess whether a BCT was overrepresented in themes completed by participants who responded to the tool by reduced HbA1c. BCT in bold have chi-square statistics corresponding to a *P* value of 0.05 or less.

Supplementary Table 16. Results from semantic analysis of questions.		
**Measure**	**n**	**Value**
Number of *abstract* questions per participant	258	6.5 (0.5)
Number of *concrete* questions per participant	260	8.1 (0.6)
Number of reported lifestyle changes in participants asking mainly *abstract* questions*	95	14.1 (1.5)
Number of reported lifestyle changes in participants asking mainly *concrete* questions**	150	9.1 (1.0)
Association between the number of *abstract* questions and change of HbA1c in all participants ***	257	-0.18 (95% CI -0.32 to -0.05)
Association between the number of *concrete* questions and change of HbA1c in all participants	259	-0.09 (95% CI -0.20 to 0.03)
Association between the number of *abstract* questions and change of HbA1c in participants using the tool as recommended	27	-0.37 (95% CI -0.71 to -0.03)
Association between the number of *concrete* questions and change of HbA1c in participants using the tool as recommended	27	0.05 (95% CI -0.17 to 0.27)
Proportion of abstract to concrete questions in participants who used the tool as recommended and *responded* with reduced HbA1c from baseline to one year****	21	1.6 (0.2)
Proportion of abstract to concrete questions in participants who used the tool as recommended and *did not respond* with similar or reduced HbA1c from baseline to one year****	6	0.5 (0.1)

*At every login, participants were given the opportunity to comment on whether their questions had resulted in lifestyle changes or not. The number of reported lifestyle changes for participants asking more abstract than concrete questions are presented. These analyses focused on data during the first year.
**The number of reported lifestyle changes in participants asking more concrete than abstract questions
***The association between the number of questions and change of HbA1c from baseline to one year was analyzed by linear regression. The beta coefficients from the regression models are reported with 95% confidence intervals.
****The proportion of abstract to concrete questions in responders (lower HbA1c at one year compared to baseline) and non-responders (similar or higher HbA1c at one year compared to baseline), respectively, in participants using the tool as recommended.
Data are means (SD); n refers to the number of participants included in the analyses.

Supplementary Table 17. Changes of biomarkers from baseline to one year used in the base case scenario. *		
**Biomarker**	**Users of the tool**	**Controls on usual care**
HbA1c (%) **	-0.55	0.12
Body mass index***	-1.09	0.23
Systolic blood pressure (mm Hg)	-4	2
Total cholesterol (mmol/l) ****	-0.05	-0.03
LDL cholesterol (mmol/l) ****	-0.01	-0.08
HDL cholesterol (mmol/l)	0.04	-0.07
Triglycerides (mmol/l) ****	-0.14	0.03
Quality-of-life	0.028	0

*Since the model is based on a cycle length of one year, the change of relevant biomarkers from baseline to one year in participants using the tool as recommended and matched controls on usual care, respectively, was included in the model

**The model equations are based on HbA1c measured in NGSP units (%). HbA1c in IFCC units (mmol/mol) was converted to NGSP (%) using the formula $HbA1c_{NGSP} = (0.915 * HbA1C_{IFCC}) + 2.15$.

***The body mass index is the weight in kilograms divided by the square of the height in meters.

****Data on total cholesterol, LDL cholesterol and triglycerides were only used in the sensitivity analysis.

Supplementary Table 18. Baseline characteristics used in the model. *		
**Demographics**	**Value**	**Data source**
Age – years	62	Study participants and matched controls
Male (%)	35	Study participants and matched controls
Diabetes duration – years	4	Study participants and matched controls
Smoker (%)	18	Study participants and matched controls
**Biomarkers**	**Value**	**Data source**
Glycated Hemoglobin (%)	6.82	Study participants and matched controls
Systolic Blood Pressure (mm Hg)	139	Study participants and matched controls
Diastolic Blood Pressure (mm Hg)	77	Study participants and matched controls
Total Cholesterol (mmol/l)	4.43	Study participants and matched controls
LDL Cholesterol (mmol/l)	2.55	Study participants and matched controls
HDL Cholesterol (mmol/l)	1.19	Study participants and matched controls
Triglycerides (mmol/l)	2.00	Study participants and matched controls
Body Mass Index	34.1	Study participants and matched controls
Heart Rate (beats per minute)	72	UKPDS[56]
White Blood Cell Count ($1x10^6$)	6.91	UKPDS 59[57]
Estimated Glomerular Filtration Rate (ml/min/1.73 m^2) **	77.8	Clinical registries[58]
**Microvascular Complications**	**Value**	**Data source**
Eye Disease		
Background Retinopathy (%)	22.5	Clinical registries[59]
Proliferative Retinopathy (%)	1.3	Clinical registries[59]
Macular Edema (%)	2.6	Clinical registries[59]
Proliferative Retinopathy and Macular Edema (%)	1.1	Clinical registries[59]
Severe Visual Loss (%) ***	0	Assumption
**Lower Extremity Disease**		
Symptomatic Neuropathy (%)	25.0	Clinical registries[60]
Peripheral Vascular Disease (%)	4.9	Clinical registries[58]
Lower Extremity Amputation (%)	0.7	Clinical registries[58]
**Kidney Disease**		
Microalbuminuria (%)	22.9	Clinical registries[58]
Macroalbuminuria (%)	4.7	Clinical registries[58]
End-stage renal disease (%) ***	0	Assumption
**Macrovascular Complications**	**Value**	**Data source**
Ischemic Heart Disease (%)	21.7	Study participants and matched controls
Myocardial Infarction (%)	11.6	Study participants and matched controls
Stroke (%)	4.3	Study participants and matched controls
Heart Failure (%)	7.2	Study participants and matched controls

*Values are means of baseline data for participants using the tool and matched controls on usual care. Since a few biomarkers included in the model were not reported in detail in the ANDIS registry, we extracted corresponding baseline data from the UK Prospective Diabetes Study (UKPDS) or clinical registries for patients with similar age, disease duration and glycemic control, in order to obtain as precise measures as possible.
**Estimated glomerular filtration rate is presented as ml per minute per 1.73 square meter of body surface area.
***Assumed to be zero from data on cohorts with similar baseline characteristics.[58,59]

**Supplementary Table 19. Treatment costs used in the model.**			
**Glucose-lowering treatment**	**Individuals (%) ***	**Average daily dose**	**Mean yearly cost (USD)****
Metformin	89	1500 mg	29
Dipeptidyl peptidase-4 inhibitors	19	100 mg sitagliptin or<br>5 mg linagliptin***	466
Sodium glucose cotransporter type-2 inhibitors	7	Dapagliflozin 10 mg or empagliflozin 25 mg***	595
Glucagon-like peptide-1 analogue	11	Liraglutide 1.2 mg or lixisenatide 20 µg***	1112
Insulin****	28	60 IU	506
Total weighted cost of treatment	-	-	**421**

*Based on data from study participants. Percentages add up to more than 100 because of co-treatment.
**Mean yearly cost per patient including consumables. Insulin costs include 1 needle, 1 test strip and 1 lancet per day. Glucagon-like peptide-1 analogue cost includes 1 needle per day.
***Approximately 50% using each compound.
****Basal neutral protamine Hagedorn (NPH) insulin was used for calculations to remain conservative.

Supplementary Table 20. Detailed results of the health economic base case scenario over 20 years. *			
	**Users of the tool**	**Patients on usual care**	**Increment**
**Survival**			
Proportion alive after 20 years – %	34.77	31.45	3.32
Remaining life expectancy – years	11.40	11.22	0.18
**Quality-adjusted life years (QALYs)**			
Life-Years	9.311	9.169	0.143
Diabetes Treatment	0.319	0	0.319
Hypoglycemia	-0.041	-0.040	-0.001
Eye Disease	-0.052	-0.058	0.006
Lower Extremity Disease	-0.492	-0.493	0.001
Kidney Disease	-0.044	-0.062	0.019
Ischemic Heart Disease	-0.171	-0.174	0.003
Myocardial Infarction	-0.034	-0.035	0.001
Stroke	-0.108	-0.116	0.008
Heart Failure	-0.138	-0.144	0.006
Age	-1.886	-1.854	-0.032
Gender	-0.415	-0.408	-0.006
Diabetes Duration	-0.128	-0.125	-0.003
Overweight	-0.549	-0.630	0.080
Total QALYs	**5.573**	**5.029**	**0.544**
**Costs (USD)**			
Diabetes Treatment	4,766**	4,609	157
Hypoglycemia	50	49	1
Eye Disease	2,143	2,507	-364
Lower Extremity Disease	10,830	10,853	-24
Kidney Disease	6,456	9,218	-2,762
Ischemic Heart Disease	3,044	3,211	-167
Myocardial Infarction	2,395	2,703	-308
Stroke	2,239	2,544	-305
Heart Failure	2,908	3,162	-254
Indirect costs (productivity loss)	16,051	16,141	-91
Total Direct Costs	34,830	38,856	-4,026
Total Costs	**50,881**	**54,997**	**-4,116**
**Incremental cost-effectiveness ratio*** **			
Direct costs			Dominant
Total costs			Dominant

*Table presents values per person during 20 years, disaggregated into components that contribute to total QALYs and costs in patients on usual care and patients using the tool in addition to usual care, respectively. Increment refers to the difference between the groups.
**Treatment costs include usual care plus tool.
***Incremental cost-effectiveness ratio is incremental total costs divided by incremental QALYs. Dominant outcome implies that the tool is more effective and cost-saving compared to usual care.

Supplementary Table 21. Summary results of the sensitivity analysis. *							
**Scenario**	**QALYs**			**Total cost (USD)**			**Break-even (USD)****
	**Tool**	**Usual care**	**Increment**	**Tool**	**Usual care**	**Increment**	
Base case	5.57	5.03	0.54	50,881	54,997	-4,116	369
1. 10-year time horizon	4.03	3.72	0.31	33,266	34,069	-803	110
2. 40-year time horizon	5.84	5.21	0.63	58,365	63,299	-4,934	411
3. Only HbA1c change	5.16	5.07	0.10	50,980	54,909	-3,930	353
4. HbA1c increase after first year in both arms	5.55	5.03	0.52	51,789	54,997	-3,208	290
5. All secondary variables	5.58	5.03	0.54	50,860	54,996	-4,136	370
6. Excluding BMI-related changes to quality of life	6.12	5.66	0.46	50,881	54,997	-4,116	369
7. Usage at least once per month, including only change in HbA1c	5.15	5.08	0.07	51,704	54,468	-2,764	251
8. Usage at least every other month, including only change in HbA1c	5.13	5.08	0.06	52,241	54,468	-2,227	204

* Table presents QALYs and costs per person during 20 years in patients on usual care and patients using the tool in addition to usual care, respectively. Increment refers to the difference between the groups. Data are shown for the base case and eight other scenarios, as detailed in the Supplementary Discussion.
**The hypothetical maximum yearly operating costs for the tool in order to remain cost-saving (“break-even”) was calculated for each scenario as total cost savings divided by total remaining life expectancy.
