# Supplementary material for: Effect of self-managed lifestyle treatment on glycemic control in patients with type 2 diabetes
Source: NPJ Digit Med. 2022 May 11;5:60. doi: 10.1038/s41746-022-00606-9 (PMC9095642; doi:10.1038/s41746-022-00606-9)
Supplement: Supplementary file 2 — Supplementary Data 1 [file 41746_2022_606_MOESM2_ESM.pdf]

(Translated from original Swedish version, approved by the regional ethics review committee 2016 no. 651.)

This study will be conducted in accordance with Good Clinical Practice (GCP)

STUDY ADMINISTRATION

Functions and responsible persons and organizations in the study are listed in Table 1.

Table 1. Study administration

Sponsor and Principal Investigator	Anders Rosengren, MD PhD Professor<br>Tel: 040-391169 / 070-5316704<br>Email: anders.rosengren@gu.se / anders.rosengren@skane.se<br>University of Gothenburg / Skåne University Hospital
Study coordinator	Birgitta Abrahamsson<br>Tel: 040-332249
Research nurses	Maria Fälemark<br>Jessica Hedin<br>Helene Ferm
Study location	CRC 62–11 Skåne University Hospital<br>Jan Waldenströms gata 35<br>20502 Malmö<br>Sweden

CONTENTS

STUDY ADMINISTRATION 1
1. SCIENTIFIC QUESTIONS 3
2. STUDY OVERVIEW 5
3. STUDY DESCRIPTION 9
3.1. Enrolment 9
3.2. Selection and recruitment 9
3.3. Consent and inclusion 10
3.4. Detailed study process 11
3.5 Discontinuation criteria 12
3.6 Travel reimbursement 13
3.7 The web tool 13
3.8 Questionnaire for evaluation of quality of life 14
4 DOCUMENTATION, SECURITY, QUALITY CONTROL 15
4.1 Documentation and archiving 15
4.2 Security 16
4.3 Insurance 16
4.4 Ethics Board and authorities 17
5 STATISTICAL ANALYSIS PLAN 18
5.1 Statistical analysis 18
5.2 Publication of data 18
6 ETHICAL CONSIDERATIONS 19
7 IMPLICATIONS 20

1. SCIENTIFIC QUESTIONS

The aim of the present study is to observe the effect of a new Internet-based lifestyle tool on metabolic control and quality of life. The Internet tool has been developed through two pilot studies conducted at Lund University. In the first pilot study, **Detailed mapping of type 2 diabetes - pilot study regarding lifestyle, social, psychological factors (EPN Lund 2014/702**), we mapped the needs for lifestyle support in patients with type 2 diabetes through interviews. This study showed a strong need for deeper lifestyle support than what healthcare offers today. In that study, we also developed motivational exercises for self-care, and the interviews showed a positive response to implementing the exercises in Internet format to make them widely accessible in a structured form. In the second pilot study, **Development of Health and lifestyle tool for type 2 diabetes (EPN Lund 2015/563)**, we implemented the motivational exercises in web form and mapped through user tests patients' preferences and perceived benefit of the Internet-based support. We have thus developed the tool in close interaction with patients to optimize usability and content from the patients' perspective.

The current study is carried out at Scania University Hospital Malmö. The university of Gothenburg and Scania University Hospital are responsible for the study. We now intend to investigate the long-term effects of the tool in patients with type 2 diabetes by an observational study, where we provide access for patients to the tool in addition to usual care and observe the natural pattern of usage and resultant outcomes.

The primary endpoints are change of HbA1c (long-term blood sugar) from baseline to twelve weeks between participants randomized to access the tool or to a wait list on usual care, and change of HbA1c from baseline to end of follow-up during the observation period between study participants using the tool as recommended and matched controls.

The secondary endpoints are

to study how the tool affects the following:

1. fasting glucose

2. body weight

3. insulin secretion measured as HOMA2-B

4. insulin sensitivity measured as HOMA2-IR

5. Cholesterol levels

6. Blood pressure

7. Fat content in the body measured by bioimpedance

8. Quality of life

9. We will also analyze the effect of the tool specifically in T2D patients with high BMI and insulin resistance.

10. The effect on HbA1c from baseline over the entire observation period for up to three years for those using the tool less than recommended year will also be studied.

2. STUDY OVERVIEW

T2D is characterized by chronically elevated blood glucose. The disease is increasing worldwide and affects more than 300 million people. It is estimated that 500 million people will have T2D by 2030. Most patients with T2D will sooner or later develop complications in the kidneys, eyes, nerves, and the cardiovascular system. Diabetes and its complications account for more than 10% of healthcare costs.

The latest guidelines from the European and American diabetes organizations (EFSD and ADA) advocate that both lifestyle interventions and pharmacological treatment of T2D should be more individualized to better prevent the serious complications. Lifestyle factors and psychological factors play a central role in disease development and disease progression (Senécal, Nouwen & White, 2000; Alam et al., 2009; Peyrot & Rubin, 2007). Strategies for self-managing are necessary in T2D and can significantly improve the patients' control over their disease and reduce the need for pharmacological treatment (Deakin et al., 2005). Lifestyle programs have been shown to reduce blood sugar by a similar magnitude to pharmacological treatment (UKPDS, Lancet 1998) and have been shown to improve self-management control and quality of life (Campbell et al., 2003; Norris et al., 2001). Improved self-management of T2D through lifestyle support can also have major societal effects by reduced long-term healthcare costs (Glasgow et al., 2011). However, a large study recently showed that only 8.7% of T2D patients report having received structured support to manage their disease (National Diabetes Audit: National Diabetes Audit 2010-2011). It has also been shown that healthcare professionals often lack knowledge about how to support patient self-management in T2D (Orme et al., 1989). A major barrier to introducing lifestyle interventions in T2D in routine healthcare has also been that these, at least in the short term, are considered costly and resource-intensive.

There is therefore a large need for new modern solutions to support patients in disease management and lifestyle changes. Web-based solutions have great potential to provide individualized, structured support, while at the same time being a cost-effective alternative that can match the growing need well (Pal et al., 2013; Murray et al., 2005; Nguyen et al., 2004). Web-based cognitive behavioral therapy has been shown to have good effects in anxiety and mild to moderate depression (https://www.beatingtheblues.co.uk/). For T2D and other lifestyle diseases, however, there is a lack of research on how web-based lifestyle support should be designed to help patients most effectively in their disease management.

Overall, the reporting of effective behavioral programs in T2D is heterogeneous, and evidence for the effectiveness of an individual theory in accordance with “one size fits all” is difficult to find (NICE, 2007; Clark, 2008). There is also limited knowledge about the effective mechanisms of programs (Murray et al., 2009). In the design of programs aimed at behavior change, it is therefore important to engage the group for which the program is designed and to base the work on a needs assessment of the group (NICE, 2007). Behavioral programs have been shown to affect the health and well-being of diabetic patients, if they provide tools for self-management and the

opportunity for feedback (Pal et al., 2013). Programs aimed at increasing patients' autonomy have also been shown to have positive, health-promoting effects and improve quality of life (Deakin et al., 2005; Marks, Allegrante & Lorig, 2005). Autonomy is a psychological concept that refers to the individual's ability and willingness to act (Anderson et al., 2000; Bandura, 1977). In a disease such as T2D, which can easily be passivating, it is important to emphasize the individual's own motivation and autonomy (Beard et al., 2010). Lifestyle diseases such as T2D should also be placed in a cultural context, where concepts such as values and meaning are included. This perspective is often neglected in behavioral interventions for T2D but may be central to increase motivation to lifestyle changes (Higgins, 2011; Bandura, 2001; Frankl, 1969). These concepts have come to play an increasingly important role in later forms of cognitive behavioral therapy (Ameli & Dattilio, 2013; Harris, 2009; Hayes, 1999). Meaning-making is also an important factor for quality of life and well-being (Melton et al., 2008; Antonovsky, 1991; Frankl, 1969).

We have recently conducted a pilot study **(Detailed mapping of type 2 diabetes pilot study regarding lifestyle, social, psychological factors, Dnr 2014/702)** which clarified the need for psychological and lifestyle support for patients with T2D. In the pilot study, patients participated in four visits that included qualitative needs interviews and the development of motivational exercises to promote healthy lifestyle changes. There was also a great interest in trying out the exercises as a new web-based treatment support. In the design of the tool, we have started from a broad theoretical base around components that affect motivation for lifestyle changes. In the design, we have addressed the possibilities and limitations identified in previous studies and introduced a unique perspective where self-reflection on health-related questions, including personal values and meaning, are central.

Design of the tool

The web-based tool will provide support for the participants in asking personally relevant questions in areas related to health, lifestyle, and diabetes management. The theoretical framework of the tool consists of a careful selection of psychological theories and models for behavior change, which address important health behaviors and areas central to good health that have been highlighted by e.g. the World Health Organization (WHO). The web-based tool is designed to help the study participants ask personally relevant questions in order to increase autonomy and disease control.

In health theoretical models for behavior change, factors that influence motivation for health behavior are central to long-term sustainable change. Health Action Process Approach (HAPA) (Schwarzer, 1992) distinguishes between motivational processes that lead to intentions and will-determined processes that lead to actual health behavior. In the motivation process, risk perception, expected outcome and perceived self-efficacy are important components in forming an intention. In the will-determined process, strategies for self-control, coping, and planning play key roles in proceeding from intention to action. Intentions are more likely to lead to active action when the patient identifies a desirable scenario and develops preparatory strategies to approach this.

The web-based tool will also emphasize the concept of self-transcendence. Social support is an important, health-promoting factor, not least when it comes to managing illness. Self-transcendence is a way of opening for deepened relationships and can thus promote the quality of social support. Self-transcendence has also significant connections to the experience of meaning in life and can thus have an impact on the study participant's quality of life and general well-being. Quality of life will therefore be an important outcome measure in the study. Patient-reported outcome measures of quality of life are central both to identify individual patient benefits and for health economic analyses. At the individual level, such measures can be used to strengthen the patient's own ability to manage disease (through empowerment), and at the population level, it can be used to motivate changes to treatment guidelines (Nilsson, E., 2014).

Patient with type 2 diabetes

Patients with type 2 diabetes is a very heterogeneous group, both from pathophysiological and psychological viewpoints. In patients with type 2 diabetes, the experience of the disease differs, where some are clearly affected by their disease states through complications, shame, anxiety, or reduced quality of life, while others experience few or no problems. However, all have an increased risk of later complications and deteriorated health because of type 2 diabetes. Support for lifestyle changes has been shown to be a preventative measure that can protect against these complications, but low risk perception, lack of confidence in one's own ability to manage the disease, or limited motivation to implement lifestyle changes are common barriers to good self-care in diabetes. Previous research has shown that autonomy plays a significant role in motivation to lifestyle changes. We will therefore focus on increasing autonomy and the sense of disease control.

Results from a previously conducted pilot study show that the patient group has varied experience of the support they receive in healthcare. Two different views were central: A) "my doctor knows me and I expect someone else to tell me what I need to do", i.e. the patients have low confidence in their own ability to manage the disease and take responsibility for lifestyle changes; B) "healthcare professionals often tell me what to do without knowing about my personal situation, and the advice I receive does not suit me", i.e. the experience of "one size fits all", which can reduce the motivation to implement lifestyle changes. In other words, there is a need to increase patients' experience of having responsibility and control over their health and to find lifestyle solutions that suit their own situation, which we intend to address in the present study.

The study participants in the study will be over 35 years of age. User engagement and retention will be an important focus in addition to the outcome variables, as we aim to observe the natural pattern of usage and resultant outcomes to make the study setting as similar as possible to the everyday context of the patients. This observational approach will increase generalizability of the results to real-life settings.

Important problems, needs or challenges that the tool intends to address

Enable long-term commitment to one's own health rather than short-term focus on temporary solutions by supporting study participants to:

- Identify diabetes-specific needs and problems
- Explore own values and meaningful activities
- Focus on own attitudes and long-term goals
- Identify own values and link them to health behaviors
- Identify obstacles and resources
- Identify meaningful life tasks and promote a self-transcendent approach
- Explore life orientation through personal questions

The tool will provide participants with research-based information on health, lifestyle, and type 2 diabetes. The tool is designed to support the study participant to actively explore health-related areas.

Guidelines for the tool

<u>Key goals</u>

• Promote exploration of health as an experience in several health-related areas.

• Support the study participant to ask personally relevant questions as a way of forming intentions to act.

• Promote retention and active use of the tool to ensure that the tool is based on the needs of the patient group and to meet sufficient statistical power.

<u>Key elements of the tool</u>

• Introduction to the concept of asking personal questions

• Relevant themes around lifestyle and T2D.

• Stimulate the study participants to ask their own questions.

• Support in letting the questions affect everyday life (through follow-up, feedback, prompt new themes)

• Focus on long-term changes / habits (less focus on short-term strategies for self-regulation).

3. STUDY DESCRIPTION

3.1. Enrolment

Inclusion criteria for participants

• Type 2 diabetes mellitus with HbA1c at 52 mmol/mol or above

• Age above 35 years

• Access to the Internet (i.e. we do not provide subscriptions or computer equipment)

• Written informed consent

Exclusion criteria

• Participation in another research study that may affect the study result

• Condition or treatment which, according to the Principal Investigator, makes participation in the study inappropriate

• Work within competing activities or participants who have interests contrary to the purpose of the study. (The reason for this exclusion criterion is that we want to prevent developers of Internet solutions for diabetes from a pure business interest from registering and copying what we have developed in an academic setting before results have been published.)

• Relation to the study through a research funder, authority, university or other body such that participants can be considered to have a special interest / gain from the study results.

• Other forms of diabetes other than type 2, such as MODY or type 1 diabetes. The reason is that these forms require different treatment than type 2 diabetes. We do not rule out that the tool later may be made available to these groups as well, but this may require special adaptations.

3.2. Selection and recruitment

Recruitment is done as follows:

1. Letters are sent to patients with type 2 diabetes registered in the ANDIS database (All New Diabetics in Scania), a project that registers the majority of newly diagnosed diabetes patients in the Scania county, aiming at better classifying diabetes (http://andis.ludc.med.lu.se/).

   When a patient in the Scania County is diagnosed with diabetes and agrees to be

part of ANDIS, blood samples are taken via regular care providers for analysis of C-peptide (measure of insulin secretion) and GAD antibodies (sign of autoimmune diabetes). Based on these data, patients are classified into different forms of diabetes based on their pathophysiology.

In several previous clinical studies, we have recruited patients from ANDIS, and we intend to use the same basic procedure here. Prior to contact, patients must be temporarily decoded to obtain personal and contact information. The study personnel send letters to patients in ANDIS with information about the study. Each patient receives an initial letter and in case of no response, a reminder letter is sent within approximately one month. If the person does not act on the reminder letter, it is assumed that the person is not interested in participating.

2. Via a dedicated registration page on the Internet for people who get to know about the study via various channels (e.g. caregivers, media, advertisements in newspapers or social media). A link to the registration page will also be available on the University of Gothenburg's web pages, and web pages with information about current research studies, such as the Swedish Diabetes Association's web portal and Diabetesportalen.se.

   The registration page contains information about the study and a form for registering interest in participation. Interested patients fill in name, email address, postal address and that they have type 2 diabetes. The postal address is needed to be able to send home additional information, and the e-mail address to be able to provide a link to the tool in included in the study. This information is stored at a server with high security. The information transfer and storage are encrypted so that unauthorized persons cannot access the information. The patients are informed that the expression of interest is not binding and only means that they receive a letter from us with additional information.

3.3. Consent and inclusion

Consent

In the consent form, the participants state his / her social security number, name, telephone number and e-mail address. The signed informed consent is stored at the study center inaccessible to unauthorized persons. Study participants who are included receive a study ID. A document that links the study ID to personal data is stored with the Investigator, inaccessible to unauthorized persons. Study ID becomes the participant’s identification on the web tool (i.e. they do not use their name there).

Inclusion

The aim of the study is to observe whether the web tool improves metabolic outcomes and quality of life and observe usage patterns. This will be important for us to be able to later recommend how the tool can best be used in healthcare.

A total of 300 patients (extra surplus will be enrolled to accommodate for dropouts) will attend study visits every 3 months during the first year and every 6 months thereafter. The purpose is to observe the development of blood glucose control over a period of up to three years. The number of patients give good statistical power to analyze changes to HbA1c. The patients also complete a questionnaire every three months.

The criteria for attending study visits are:

a) Living in the county of Scania, i.e. in reasonable vicinity of the study center. Participants receive travel reimbursement. If participants who live outside the county want to attend study visits, they are welcome to do so but are informed that they cannot claim travel reimbursement.
b) HbA1c at 52 mmol/mol or above
c) Type 2 diabetes

3.4. Detailed study process

After signed written informed consent, the following procedures are conducted:

1. Individuals who are interested in participating will be contacted by study staff via email or telephone to book a time for a first visit. Patients are then also asked about their current HbA1c value. If current HbA1c is below 52 mmol/mol the patient will not attend any study visit. If current HbA1c is 52 or above or is unknown to the patient, he/she will be invited for a first visit.

2. Prior to blood sampling, patients should be fasting. They are instructed not to exercise strenuously or to drink alcohol the day before and not to use nicotine the same day, as it may affect blood tests. The following is done during the visit:

    a. ID is checked
    b. HbA1c is analyzed on-site. If 52 mmol/mol or above, patients proceed to the next steps.
    c. Length and weight are checked
    d. Blood pressure and heart rate are measured
    e. Bioimpedance measurement is done to measure fat distribution
    f. Fasting venous blood samples are taken as follows (total blood volume approx. 23 ml)
        i. HbA1c
        ii. Fasting glucose
        iii. C-peptide (measure of insulin secretion and used to calculate HOMA)
        iv. Total cholesterol, HDL, LDL, triglycerides

3. They are randomized to access the tool directly or to wait (ratio 1:1)

4. Study participants receive an email with a login link that takes them to the study account where they complete the questionnaire.

5. Those who have been randomized to immediate access to the tool will enter the tool directly after completing the questionnaire. They use the tool on their own from home.

6. After 3 months, the study participants arrive for the next physical visit (including those randomized to the waiting list). The same samples are taken as at the first visit. Those who received immediate access to the tool will continue as before and those who have been on a waiting list will receive a new email message after the visit with a login link that takes them into the tool.

7. When the study participants have been observed for a year, which is the primary time point for assessment of study variables, they will be given the opportunity to continue according to the procedure above.

3.5 Discontinuation criteria

Study participants can discontinue at any time without giving a specific reason and without any consequences for his/her future treatment. Based on our pilot studies we recommend that the tool is used at least every other week. Study participants will not be excluded if they have lower frequency of use, as we are interested in studying the natural pattern of usage. It may for example be that patients want to pause their use of the tool during some periods and return if they get problems with health. Those who have less than 6 logins per 3-month period will however not be the included in data analysis of the primary variable or the questionnaire data, as they are not defined as “active users”.

The study may be discontinued for a participant if study instructions are not adhered to. This may include entering unserious, offensive or illegal data when they complete themes on the tool, distributing copyright-protected material from the tool without explicit approval or in other ways damage the purpose of the study.

Study participants can contact the study team if they have any issues while using the tool. Medical issues will be referred to ordinary healthcare if necessary.
Participants can request to have parts or all of their data removed from the tool. Data that have been included in analyses or scientific publications can however not been removed from such analyses.

3.6 Travel reimbursement

Participants receive travel reimbursement after study visits. No other reimbursement is paid. Participants use the tool via their own computer, tablet or mobile phone.

3.7 The web tool

The tool is designed to help the study participant to ask personally relevant questions to increase their autonomy, disease control and promote daily routines that affect diabetes and quality of lie. The approach intends to stimulate reflection to support long-term behavioral change (Senay et al.).

Participants get access to a range of themes based on research material that deepens their insights on a specific topic, such as diet, exercise, time prioritization, life balance, self-awareness, relationships, social support. Participants choose their themes freely.

Themes have a structure where study participants make self-assessments through different tests and exercises and receive algorithm-based feedback. It can, for example, be about their physical activity during a typical week, about their eating behavior or how they prioritize their time. They then read texts to provide further information and inspiration. These texts are written within the study team and references to further literature are provided for those study participants who want to know more.

After completing a theme, participants ask themselves a question about something that is important in light of the theme they have just completed. We give example questions to help the study participants, but they can also formulate their own question. From the previous pilot studies of the tool we have observed that patients ask a wide range of questions and become gradually more proficient at writing relevant questions for themselves. We also have instructional videos to help formulate effective questions.

After the study participants have written their personal question, they reflect on the question in everyday life to explore different options and try different solutions for lifestyle changes. When participants return to the tool (within two weeks is recommended), they evaluate whether the question has led to thoughts or concrete steps. They then choose the next theme, and after this theme they can choose to keep their previous question or write a new one, inspired by the insights they have gained in the theme.

The questions are used as a basis to stimulate goal-directed behavior (Senay et al.) and a more open attitude to lifestyle changes. Through the different themes, we help the study participants to focus on relevant problems and give them a framework for formulating personal questions on this problem. They receive instructions on how to write effective questions, e.g. that the question should be in how-form, i.e. focused on attitude and behavior (e.g. How can I avoid snacking when I get stressed?), rather than

in what-form (e.g. What is most healthy of x and y?). They do not receive a direct answer to the question from the study team or other healthcare professionals, but use the question as a support to take a new direction and set personal behavioral goals.

The study participants use the tool on their own from home via computer, tablet, or mobile phone. They can always contact the study staff by telephone or email for technical support. For medical issues, they are referred to regular healthcare providers.

3.8 Questionnaire for evaluation of quality of life

The study participants, regardless of whether they are on the wait list or use the tool, will be asked to complete a questionnaire every three months. The questionnaire is completed via their personal study account and is separated from other exercises on the tool. The answers are stored electronically on a secure server with encrypted data transfer. The study participants are informed that the study staff does not see what they answer to the questionnaires, and the answers are stored in a coded form using study ID. The questionnaire contains the following parts:

• Background information on age, gender, smoking, duration of diabetes, diabetes medication, and diabetic complication.

• EQ-5D-5L, a frequently used scale for measuring quality of life as a basis for health economic calculations and future care recommendations.

4 DOCUMENTATION, SECURITY, QUALITY CONTROL

4.1 Documentation and archiving

On the registration page, interested study participants fill in their name, postal address, and e-mail address. Data is stored on a secure server in Sweden.

On the consent form, the study participants fill in their name, social security number, telephone number and email address. The consent forms are archived at the study centre.

Study participants receive a study ID from 1001 to 1999. The code list of personal ID linked to study ID is stored at the study center, inaccessible to unauthorized persons. The code list contains the following information: name, social security number, telephone number, email address and study ID. Study documents will be archived at least 10 years after end of study.

Included study participants receive an email with a link to login. They log in with their email address and a password that they decide themselves. They first get access to the questionnaire. After completing this, they are informed of whether have been randomized to the wait list or to access the tool immediately. Those who have been randomized to the wait list receive a new email after 3 months with a link that takes them to the questionnaire again and then access to the tool.

The data collected via the tool is stored on a secure server. On this server is study ID, email address and password, as well as data collected via the tool. Data will be exported for analysis as a text file. Exported data are only tied to study ID (not email address), and only coded data, based on study ID, are used for analyses. Only the Investigator or delegated staff have access to data. The data that the study participants enter on the tool concern personal reflections, answers to exercises, personal questions, and evaluation of these questions.

All contacts with the study participants at study visits are registered in a case report form (CRF) that is prepared specifically for the current study.

The study participants do not have access to each other's accounts or data, but use the tool on their own. Entered data from the study participants may be displayed on the tool as an example to provide help on how the tool can be used. However, we ensure that all such information is completely de-identified and cannot be linked to any person through specific details.

The study participant can request to see their own data and to have all or parts of the collected data deleted at any time. All data from the study that is presented or published will be anonymized, i.e. study ID is not communicated, and we remove any identifiable information that the study participants have entered.

4.2 Security

All data are stored on servers. The system is backed up once a day to be able to recreate if necessary (for e.g. in the event of a server error).

Data transfer is encrypted using https. Only delegated study staff and database managers at the web agency (Happiness AB, Sweden) who assist with technical development and the server manager (Cloudnet AB, Sweden) have access to data. Happiness and Cloudnet do not handle individual data but have access to the database to be able to maintain the application. The University of Gothenburg, which provides the tool, have established an agreement with Happiness and Cloudnet that binds them to handle personal data with confidentiality. Only specific technicians have physical access to the physical server spaces.

Each study participant has his/her own personal account to which no other participant has access. If study participants forget their password, they can request a new password via a link.

The study team has the required experience of methods and procedures. The study team and the principal investigator have conducted several large studies, for example since 2013 **"Detailed mapping of type 2 diabetes"** (Dnr 2013/84), **"Randomized study with yohimbine in type 2 diabetes patients with a genetic risk variant for ADRA2A"** (Dnr 2011/587; EUDRA-CT 2010-018604-85) and **“Effect of broccoli in type 2 diabetes”** (Dnr 2015/395). The study team has also led the two pilot studies on the web-based tool, **"Detailed mapping of type 2 diabetes pilot study regarding lifestyle, social, psychological factors"** (EPN Dnr 2014/702) and **"Health and lifestyle development web tool for type 2 diabetes"** (EPN Dnr 2015/563). Both principal investigator and research nurses have completed courses in Good Clinical Practice. Principal investigator Anders Rosengren is a physician at the Department of Endocrinology, Skåne University Hospital and professor at the University of Gothenburg (and formerly at Lund University). Anders Rosengren has been responsible for the above-mentioned studies and is also clinically responsible for the ANDIS cohort.

The risks of the study are small. Blood samples are taken according to routine procedures. No other invasive measures are taken.

The study team has no responsibility for the treatment of the study participants. The study participants continue to be followed by their regular caregiver during the study and are encouraged to inform the caregiver if major changes in lifestyle are made. Caregivers cannot access the study participants' data on the tool (unless the participant himself / herself chooses to show this). However, the caregiver can, via the common clinical laboratory system, access the results of the participants' blood samples.

The study team does not examine what individual participants write on the tool but will analyze overall patterns of data. The study team can therefore not take any responsibility to follow up entered data unless contacted directly by study participants with requests for help.

4.3 Insurance

The study participants are insured through the Patient Injuries Act.

4.4 Ethics Board and authorities

The study can start when written approval from the Regional Ethics Review Board (REPN) is available. If significant changes to the study protocol are made after approval, an amendment to the protocol ("Amendment") must be written and sent to REPN for approval before this change can be implemented. All correspondence with REPN is archived in the sample folder intended for the study.

5 STATISTICAL ANALYSIS PLAN

5.1 Statistical analysis

The two primary endpoints are the change of HbA1c (long-term blood sugar) from first to second visit (time interval 60-120 days) between randomization groups, and from baseline to one year in participants using the tool as recommended, compared with matched controls.

EQ-5D-5L will be used to measure quality of life and will together with biological effect variables be used for a cost-effectiveness analysis of the tool.

Matched controls will be selected from the ANDIS cohort in an anonymous way based on study ID in ANDIS.

The primary and secondary endpoints will be analyzed by Student's t-tests. The influence of high BMI and insulin resistance on HbA1c will be analyzed by a linear model with an interaction term.

The study is dimensioned for 300 participants (a surplus will be recruited to accommodate for dropouts). The number is based on that 142 patients are needed to observe a 2 mmol/mol decrease in HbA1c with 80% power and alpha=0.05 (with standard deviation 6 mmol / mol). All participants not lost to follow-up between first and second visit will be included.

If participants change medication during the long-term observation period, then data from the last visit with unchanged medication is used for analysis. A total of 24 participants using the tool as recommended and 48 matched controls are needed to detect a 5 mmol/mol decrease in HbA1c with 80% power at alpha=0.05 (with standard deviation 7 mmol/mol for the change of HbA1c per year as estimated from ANDIS patients).

5.2 Publication of data

Study data will be continuously reported publicly in international medical journals, at scientific conferences, in various patient forums and in the media. Only deidentified data is used in these contexts.

6 ETHICAL CONSIDERATIONS

The study is carried out in accordance with the protocol, applicable laws and regulations, the principles of the GCP and the Declaration of Helsinki.

In our opinion, the benefit of participating in the study outweighs the risks. The participants are recommended to use the tool at least biweekly but decide themselves how active they want to be on the web-tool, in order to capture the natural pattern of usage. All data entry is voluntary. Those study participants who after a while do not want to use the tool can simply refrain from logging in or request a formal withdrawal.

Participants are not exposed to any medical risks. As a patient, gaining increased insights and knowledge about your situation and disease is in most cases perceived as positive, which our pilot studies have also confirmed. The pilot studies also indicated that the tool was an opportunity for increased self-awareness.

The study may benefit other patients by evaluating a new comprehensive, patient-centered lifestyle support.

Study participants will work to strengthen their motivation and deepen their understanding of how they prioritize and how their everyday decisions affect health. The participants only fill in information for their own sake, i.e. the caregiver does not see what they have filled in. The study team does not provide individual health advice on the portal but provide general advice and information as well as a framework so that study participants can formulate and follow up their personal questions and goals.

The tool is designed to be easy to use and requires no advanced computer skills. To facilitate use, there are also extensive instructions. Participants also can contact the study staff to get technical support. In these cases, individual medical advice will not be provided, but if there is a need for medical support or in-depth discussions, participants will be referred to regular care providers.

The reflections and data that the study participants fill in on the tool will contain sensitive personal information. Therefore, we have taken several measures to keep data security as high as possible, including secure servers and encrypted transfer. The participant can request to see their data and delete parts or all the data from the tool.

Unfortunately, the study excludes participants who do not have access to the web, which can be negative for, for example, older or socially vulnerable groups. This is a general problem for patient-centered web-based medicine that has been discussed, for example, in the EU, where some countries in Eastern Europe have relatively low web access. The consensus from the EU Commission has so far been (e.g. the EU's eHealth Strategy 2011) to strongly advocate research and development on web support for patients as 1) the potential for these tools is great as a scalable and cost-effective future complement to routine care that increases patients' influence and 2) Internet access is constantly increasing, even among the elderly and the socially disadvantaged.

Overall, we believe that the benefits of the study outweigh the risks. The study can lead to a better understanding of type 2 diabetes, knowledge that in the long run can benefit many patients for both treatment and prevention.

7 IMPLICATIONS

The need for relevant, health-promoting lifestyle tools for type 2 diabetes can be assumed to continue to increase as the disease increases in large parts of the world and traditional healthcare rarely has the resources required to provide structured, individualized care that addresses lifestyle and psychological factors. Lifestyle factors play a central role in the development and progression of type 2 diabetes. Strategies for managing one's own health can significantly improve control over the disease and reduce the need for medical treatment. It could also prevent the development of the disease in non-diabetic individuals. Unfortunately, this important area is still relatively unexplored. A major barrier to introducing face-to-face lifestyle interventions for type 2 diabetes in routine medical care has been that these, at least in the short term, are considered costly and resource-intensive. There is therefore an extensive need for new modern solutions. Web-based solutions have great potential to provide the opportunity for individualized, structured support, while at the same time being a cost-effective alternative that addresses the growing need. One problem today is that we do not know how to best design these new patient-centered tools. Development in this area is rapid but are led largely by commercial actors whose “health apps” often lack research support. It is therefore important that scientifically based eHealth solutions are developed in academia and healthcare. This study will be an important step in evaluating an academically developed tool for promoting health. The study is innovative in that we focus on deeper values and self-reflection to increase the quality of life, self-management and motivation and making lifestyle changes in line with the patient’s own situation.

8. REFERENCES

Alam, R., Sturt, J., Lall, R., Winkley, K. An updated meta-analysis to assess the effectiveness of psychological interventions delivered by psychological specialists and generalist clinicians on glycaemic control and on psychological status. Patient education and counseling 75 (2009) 25-36

Ameli, M. & Dattilio, F.M. Enhancing cognitive behavior therapy with logotherapy: Techniques for clinical practice. Psychotherapy 50 (3) (2013) 387-391

Anderson, R.M., Funnell, M.M, Fitzgerald, J.T., Marrero, D.G. The Diabetes Empowerment Scale. A measure of psychosocial self-efficacy. Diabetes Care 23 (2000) 739–743

Antonovsky, A. (1991). Hälsans mysterium. Stockholm: Natur och kultur.

Bandura, A. Self-efficacy: Toward a unifying theory of behavioral change. Psychological Review 84 (2) (1977) 191-215

Bandura, A. Social Cognitive Theory: An agentic perspective. Annual Reviews Psychology 52 (2001) 1-26

Beard, E., Clark, M., Hurel, S., Cooke, D. Do people with diabetes understand their clinical marker of long-term glycemic control (HbA1c levels) and does this predict diabetes self-care behaviours and HbA1c? Patient Education and Counseling 80 (2010) 227–232

Bryman, A. (2012). Social Research Methods. 4ed. New York: Oxford University Press.

Campbell, R., Pound, P., Pope, C., Britten, N., Pill, R., Morgan, M., Donovan, J. Evaluating meta-ethnography: a synthesis of qualitative research on lay experiences of diabetes and diabetes care. Social Science & Medicine 56 (2003) 671–684

Clark, M. Diabetes self-management eduction: A review of published studies. Primary care diabetes 2 (2008) 113-120

Corbin, J. & Morse, J. The unstructured interactive interview: Issues of reciprocity and risks when dealing with sensitive topics. Qualitative Inquiry 9 (3) (2003) 335-354

Deakin, T.A., McShane, C.E., Cade, J.E., Williams, R. Group based training for self-management strategies in people with type 2 diabetes mellitus. Cochrane Review (2005)

Franklin, C., Trepper, T.S., McCollum, E.E., Gingerich, W.J. (2011). Solution-focused brief therapy: A handbook of evidence-based practice. New York: Oxford University Press.

Glasgow, R.E., Kurz, D., King, D., Dickman, J.E., Faber, A.J., Halterman, E., Woolley, T., Toobert, D.J., Strycker, L.A., Estabrooks, P.A., Osuna, D., Ritzwoller, D. Twelve-month outcomes of an Internet-based diabetes self-management support program. Patient Education and Counseling 87 (2012) 81–92

Harris, R. Act made simple (2009). Oakland: New Harbinger Publications.

Hayes S.C, Strosahl, K., Wilson, K. (1999) Acceptance and Commitment Therapy: An experiential approach to behavior change. Guilford Press.

Higgins, E.T. (2011). Beyond pleasure and pain: How motivation works. New York: Oxford University Press.

King, M., Speck, P., Thomas, A. The effect of spiritual beliefs on outcome from illness Social Science & Medicine 48(9) (1999) 1291

Lancaster, G.A., Dodd, S., & Williamson, P.R. Design and analysis of pilot studies: recommendations for good practice. Journal of Evaluation in Clinical Practice 10 (2) (2002) 307–312

Marks, R., Allegrante, J.P., & Lorig, K. A review and synthesis of research evidence for self-efficacy-enhancing interventions for reducing chronic disability: implications for health education practice (part I). Health promotion practice 6 (1) (2005) 37-43

Marks, R., Allegrante, J.P., & Lorig, K. A review and synthesis of research evidence for self-efficacy-enhancing interventions for reducing chronic disability: implications for health education practice (part II). Health promotion practice 6 (2) (2005) 148-156

Maxwell, J.A. Qualitative research design. Chapter 7: Designing a qualitative study (2005). SAGE publications.

Murray, E., Burns, J., See Tai, S., Lai, R., Nazareth, I. Interactive health communication applications for people with chronic disease. Cochrane Review (2009)

National Diabetes Audit: National Diabetes Audit 2010-2011

Nguyen, H.Q., Carrieri-Kohlman, V., Rankin S.H., Slaughter, R., Stulbarg, M.S. Internet-based patient education and support interventions: a review of evaluation studies and directions for future research. Computers in Biology and Medicine 34 (2004) 95-112

Norris, S.L., Engelgau, M.M., & Narayan, K.M.V. Effectiveness of Self-Management Training in Type 2 Diabetes: A systematic review of randomized controlled trials. Diabetes Care 24(3) (2001) 561-587

National Institute for Health and Clinical Excellence. Behaviour change at population, community and individual levels. (2007)

Orme C.M., Binik, M., & Yitzchak, M. Consistency of adherence across regimen demands. Health Psychology 8(1) (1989) 27-43

Pal, K., Eastwood, S.V., Michie, S., Farmer, A.J., Barnard, M.L., Peacock, R., Wood, B., Inniss J.D., Murray, E. Computer-based diabetes self-management interventions for adults with type 2 diabetes mellitus. Cochrane Review (2013)

Patton, M. (1990) Qualitative evaluation and research methods (pp. 169-186) Beverly Hills: Sage.

Peyrot, M. & Rubin, R.R. Behavioral and psychosocial interventions in diabetes. A conceptual review. Diabetes Care 30(10) (2007)

Senécal C., Nouwen, A., & White, D. Motivation and dietary self-care in adults with diabetes: Are self-efficacy and autonomous self-regulation complementary or competing constructs? Health Psychology 19 (5) (2000) 452-457

Sullivan M et al., Soc Sci Med 1995; 41.

UKPDS (1998). Intensive blood-glucose control with sulphonylureas or insulin compared with conventional treatment and risk of complications in patients with type 2 diabetes (UKPDS 33). UK Prospective Diabetes Study (UKPDS) Group. Lancet 352, 837-853.

Williams, G. C., Freedman, Z.R., & Deci, E. L. Supporting autonomy to motivate glucose control in patients with diabetes. Diabetes Care 21 (1998) 1644-1651.

World Values Survey 2010; http://www.worldvaluessurvey.org

WHOQOL-SRPB Users Manual, Scoring and Coding for the WHOQOL

SRPB Field-Test Instrument. 2002. Geneva: Department of Mental Health and Substance Dependence, World Health Organization.
