# Supplementary material for: Effect of self-managed lifestyle treatment on glycemic control in patients with type 2 diabetes
Source: NPJ Digit Med. 2022 May 11;5:60. doi: 10.1038/s41746-022-00606-9 (PMC9095642; doi:10.1038/s41746-022-00606-9)
Supplement: Supplementary file 3 — Reporting Summary [file 41746_2022_606_MOESM3_ESM.pdf]

nature portfolio

Corresponding author(s): Anders Rosengren
Last updated by author(s): Nov 26, 2021

Reporting Summary

Nature Portfolio wishes to improve the reproducibility of the work that we publish. This form provides structure for consistency and transparency in reporting. For further information on Nature Portfolio policies, see our Editorial Policies and the Editorial Policy Checklist.

Statistics

For all statistical analyses, confirm that the following items are present in the figure legend, table legend, main text, or Methods section.

n/a	Confirmed	
☐	☒	The exact sample size (*n*) for each experimental group/condition, given as a discrete number and unit of measurement
☐	☒	A statement on whether measurements were taken from distinct samples or whether the same sample was measured repeatedly
☐	☒	The statistical test(s) used AND whether they are one- or two-sided<br>*Only common tests should be described solely by name; describe more complex techniques in the Methods section.*
☐	☒	A description of all covariates tested
☐	☒	A description of any assumptions or corrections, such as tests of normality and adjustment for multiple comparisons
☐	☒	A full description of the statistical parameters including central tendency (e.g. means) or other basic estimates (e.g. regression coefficient) AND variation (e.g. standard deviation) or associated estimates of uncertainty (e.g. confidence intervals)
☐	☒	For null hypothesis testing, the test statistic (e.g. *F*, *t*, *r*) with confidence intervals, effect sizes, degrees of freedom and *P* value noted<br>*Give P values as exact values whenever suitable.*
☒	☐	For Bayesian analysis, information on the choice of priors and Markov chain Monte Carlo settings
☒	☐	For hierarchical and complex designs, identification of the appropriate level for tests and full reporting of outcomes
☒	☐	Estimates of effect sizes (e.g. Cohen's *d*, Pearson's *r*), indicating how they were calculated

Our web collection on statistics for biologists contains articles on many of the points above.

Software and code

Policy information about availability of computer code

Data collection	Data were collected using the treatment tool, which is based on Drupal ver. 8.
Data analysis	Statistical analyses were performed using SPSS (v25).

For manuscripts utilizing custom algorithms or software that are central to the research but not yet described in published literature, software must be made available to editors and reviewers. We strongly encourage code deposition in a community repository (e.g. GitHub). See the Nature Portfolio guidelines for submitting code & software for further information.

Data

Policy information about availability of data

All manuscripts must include a data availability statement. This statement should provide the following information, where applicable:

- Accession codes, unique identifiers, or web links for publicly available datasets
- A description of any restrictions on data availability
- For clinical datasets or third party data, please ensure that the statement adheres to our policy

Data requests should be submitted to the corresponding author for consideration. Access to anonymized data may be granted following review. The trial protocol is appended to the article.

Field-specific reporting

Please select the one below that is the best fit for your research. If you are not sure, read the appropriate sections before making your selection.

☒ Life sciences ☐ Behavioural & social sciences ☐ Ecological, evolutionary & environmental sciences

For a reference copy of the document with all sections, see nature.com/documents/nr-reporting-summary-flat.pdf

Life sciences study design

All studies must disclose on these points even when the disclosure is negative.

Sample size	The study had two primary endpoints: the change of HbA1c from baseline to twelve weeks between randomization groups and from baseline to one year in participants using the tool as recommended compared to matched controls on usual care.<br>The sample size, 142 participants in each of the randomization groups, was calculated to ensure at least 80% power at alpha=0.05 to detect a significant difference between the groups, assuming that the true treatment effect of the tool is 2 mmol/mol over 12 weeks with a standard deviation of 6 mmol/mol for the change of HbA1c.<br>For the second primary variable we needed 24 participants using the tool as recommended and 48 matched controls to have 80% power at alpha=0.05 to detect a significant difference between the groups, assuming that the true treatment effect of the tool is 5 mmol/mol with a standard deviation of 7 mmol/mol for the change of HbA1c. (The standard deviation for the change of HbA1c per year was estimated from observations of patients in ANDIS with baseline HbA1c ≥ 52 mmol/mol).
Data exclusions	Those lost to follow-up between first and second visit were not included in the randomization analysis. All other participants were included, independent of medication change or frequency of using the tool.<br>If participants were lost to follow-up or changed glucose-lowering medicines during the follow-up period, then data from the last visit with unchanged medicines was used for analysis.
Replication	Findings were consistent at 12 weeks, one year and at the end of follow-up (average 730 days) at different usage patterns. The secondary variable outcomes also support the primary outcomes, showing broad metabolic improvements, including fasting glucose, insulin resistance, insulin secretion, body weight and fat mass.<br>To investigate the likelihood that the observed changes represented only naturally occurring fluctuations in glucose control, we analyzed the pattern of HbA1c progression in 13,561 patients with T2D in the ANDIS cohort during three-year frames. The relative number of patients with sustained HbA1c improvement was significantly higher in those exposed to the tool than what would be expected by chance.<br>The characterization of MOD was verified in a separate cohort and the pronounced effect in MOD was evident both during randomization and the long-term observational assessment.
Randomization	After the first visit, participants were randomized via a web-based system to have immediate access to the tool or wait for twelve weeks. In this manner, allocation was concealed to both participants and study personnel at the first visit. The generation of the random sequence, participant enrolment by study personnel, and the web-based system for allocation to randomization groups were clearly separated.
Blinding	The study is open-label since treatment was based on a digital tool where placebo was not feasible.

Reporting for specific materials, systems and methods

We require information from authors about some types of materials, experimental systems and methods used in many studies. Here, indicate whether each material, system or method listed is relevant to your study. If you are not sure if a list item applies to your research, read the appropriate section before selecting a response.

Materials & experimental systems

n/a	Involved in the study	
☒	☐	Antibodies
☒	☐	Eukaryotic cell lines
☒	☐	Palaeontology and archaeology
☒	☐	Animals and other organisms
☐	☒	Human research participants
☐	☒	Clinical data
☒	☐	Dual use research of concern

Methods

n/a	Involved in the study	
☒	☐	ChIP-seq
☒	☐	Flow cytometry
☒	☐	MRI-based neuroimaging

Human research participants

Policy information about studies involving human research participants

Population characteristics	Individuals with type 2 diabetes (both female and men) older than 35 years with HbA1c ≥ 52 mmol/mol were eligible for enrolment, independent of disease duration and treatment.
Recruitment	Study information was provided via letters to patients in the ANDIS registry or via advertisements and participants were

Recruitment	recruited by self-selection. We undertook several measures to ensure the results are not due to self-selection bias. These include: confounder adjustment using propensity scores, comparison with spontaneous glucose fluctuations in other cohorts, exposure-response analyses, and semantic analyses using machine learning showing that the metabolic improvement was coupled with exposure to the reflective elements of the tool.
Ethics oversight	The regional ethics review committee, Gothenburg Sweden

Note that full information on the approval of the study protocol must also be provided in the manuscript.

Clinical data

Policy information about clinical studies

All manuscripts should comply with the ICMJE guidelines for publication of clinical research and a completed CONSORT checklist must be included with all submissions.

Clinical trial registration	ClinicalTrials.gov identifier: NCT04691973
Study protocol	The study protocol is appended with the paper.
Data collection	Participants attended study visits in Malmö, Sweden, every 3-6 months for blood sampling and physical examination but received no counselling or lifestyle advice from the study personnel. Technical problems were referred to a study coordinator.
Outcomes	The primary study variable was HbA1c in blood assessed by Capillary 3 TERA Haemoglobin A1c Kit. Secondary variables included: body weight, fat and muscle mass (assessed by bioimpedance), fasting blood glucose, total cholesterol, LDL cholesterol, HDL cholesterol, triglyceride levels (all blood analytes measured on Cobas, Roche Diagnostics, Mannheim, Germany), blood pressure, and homeostasis model assessment-2 estimates of insulin resistance (HOMA2-IR) and beta-cell function (HOMA2-B).
